# Fatty acid fingerprints in bronchoalveolar lavage fluid and its extracellular vesicles reflect equine asthma severity

**DOI:** 10.1038/s41598-023-36697-x

**Published:** 2023-06-17

**Authors:** Nina Höglund, Petteri Nieminen, Anne-Mari Mustonen, Reijo Käkelä, Sylvain Tollis, Ninna Koho, Minna Holopainen, Hanna Ruhanen, Anna Mykkänen

**Affiliations:** 1 Department of Equine and Small Animal Medicine, Faculty of Veterinary Medicine, University of Helsinki, 00014 Helsinki, Finland; 2 School of Medicine, Faculty of Health Sciences, Institute of Biomedicine, University of Eastern Finland, 70211 Kuopio, Finland; 3 Department of Environmental and Biological Sciences, Faculty of Science, Forestry and Technology, University of Eastern Finland, 80101 Joensuu, Finland; 4 Helsinki University Lipidomics Unit, HiLIPID, Helsinki Institute of Life Science, HiLIFE, and Biocenter Finland, 00014 Helsinki, Finland; 5 Molecular and Integrative Biosciences Research Programme, Faculty of Biological and Environmental Sciences, University of Helsinki, 00014 Helsinki, Finland

**Keywords:** Asthma, Extracellular signalling molecules, Fatty acids, Translational research, Inflammation

## Abstract

Equine asthma (EA) is an inflammatory disease of the lower airways driven by mediators released from cells. Extracellular vesicles (EVs) are vehicles for lipid mediators, which possess either pro-inflammatory or dual anti-inflammatory and pro-resolving functions. In this study, we investigated how the respiratory fatty acid (FA) profile reflects airway inflammatory status. The FA composition of bronchoalveolar lavage fluid (BALF), BALF supernatant, and bronchoalveolar EVs of healthy horses (n = 15) and horses with mild/moderate EA (n = 10) or severe EA (SEA, n = 5) was determined with gas chromatography and mass spectrometry. The FA profiles distinguished samples with different diagnoses in all sample types, yet they were insufficient to predict the health status of uncategorized samples. Different individual FAs were responsible for the discrimination of the diagnoses in different sample types. Particularly, in the EVs of SEA horses the proportions of palmitic acid (16:0) decreased and those of eicosapentaenoic acid (20:5n-3) increased, and all sample types of asthmatic horses had elevated dihomo-*γ*-linolenic acid (20:3n-6) proportions. The results suggest simultaneous pro-inflammatory and resolving actions of FAs and a potential role for EVs as vehicles for lipid mediators in asthma pathogenesis. EV lipid manifestations of EA can offer translational targets to study asthma pathophysiology and treatment options.

## Introduction

Equine asthma (EA) is an inflammatory disease of the lower airways promoted by inflammatory modulators, such as lipid mediators^1,2^. EA is prevalent in stabled horses around the world^3,4^. It is classified by severity into mild/moderate (MMEA) and severe (SEA) forms, of which MMEA cases show poor performance and respiratory symptoms when exercised, while SEA cases suffer from respiratory distress also at rest. Inflammation in the lower airways can be triggered by various types of inhalable particles, such as molds, fungi, and dust, leading to immune cell infiltration in the airway lumen, bronchoconstriction, and remodeling of the airways^5^.

Cells secrete extracellular vesicles (EVs) by budding from the plasma membrane or by releasing them from multivesicular bodies, and they are taken up by recipient cells by endocytosis or fusion with the cell surface^6,7^. EV cargo, which includes miRNAs, lipids, and proteins, is protected from degradative enzymes and chemical substances by the lipid bilayer and a protein corona^8^. Earlier studies suggest that the number of EVs and their lipid content change in chronic inflammatory conditions, such as asthma, and EVs could be considered vehicles for bioactive lipids and their derivatives^1,9^. The delivered EV cargo can regulate cellular protein synthesis, metabolic pathways, and signaling and, thus, EV therapies raise hopes to modulate the progression of inflammatory diseases^10,11^.

Inflammatory responses that remain self-limiting have acute, class switch, and resolution phases. During the acute phase, fatty acids (FAs) hydrolyzed from body lipids are converted to pro-inflammatory lipid mediators^12^. The initial dominance of pro-inflammatory prostaglandins and leukotrienes is later class-switched to anti-inflammatory eicosanoids and specialized pro-resolving mediators (SPMs). The various lipid mediators are metabolites of polyunsaturated FAs (PUFAs) and, thus, the availability of different PUFA precursors modulates the tissue lipid mediator profile and immune responses^13^. While the SPMs derived from n-3 PUFAs have anti-inflammatory and pro-resolving properties, the roles of the n-6 PUFA-derived mediators are predominantly pro-inflammatory, however, n-6 PUFA derivatives with anti-inflammatory and pro-resolving activity are also known^14^. If the tissue PUFA composition and the delicate balance of lipid mediators are disrupted, the resolution of acute inflammation fails, and pathological processes with chronic inflammation ensue. Human and equine asthma therapies have taken advantage of the anti-inflammatory effects of PUFAs, which control the resolution by affecting inflammatory cell activity and cytokine balance^15,16^. Oral supplements of PUFA combinations have been shown to increase the body reservoir of anti-inflammatory PUFAs in horses^17,18^ and are recommended as a part of EA therapy^19^.

Lipids are the main components of pulmonary surfactant, which reduces the tension in the alveolar epithelium and thereby prevents the alveoli from collapsing. Furthermore, surfactant contributes to the immune defense against pathogens and allergens. Surfactant lipids of mammals, including horses, mainly consist of phospholipids, predominantly saturated phosphatidylcholines harboring palmitic acid (16:0)^20,21^. Alterations in lipid and protein composition of pulmonary surfactant facilitate the progression of airway dysfunction by contributing to the function of alveoli, airway smooth muscle, and regulation of inflammatory cell activity^13,22^. Recent research has revealed alterations in the surfactant lipids in horses with naturally occurring MMEA and SEA compared to controls^23^. However, to the best of our knowledge, the detailed FA compositions of different bronchoalveolar lavage fluid (BALF) components and BALF EVs have not been previously studied.

This study aims to close this gap by comparing the FA composition of BALF and its components between horses with EA and healthy horses in order to identify FA fingerprints characteristic of EA, and to investigate the role of BALF EVs as carriers of FAs. We hypothesized that (i) bronchoalveolar EVs would serve as vehicles for FAs, and that (ii) the EV FA profiles would differ between horses with asthma and control horses and reflect the inflammatory status of the animal.

## Materials and methods

### Animals, sample collection, and preparation

Privately owned horses (n = 12) and ponies (n = 3) with naturally occurring EA, and healthy control horses (n = 13) and ponies (n = 2) were enrolled in this prospective clinical case–control study conducted at the Faculty of Veterinary Medicine, University of Helsinki. The animal experiment was approved by the Project Authorisation Board (ESAVI/3285/2020), and all experiments were performed in accordance with EU regulations and ARRIVE guidelines^24^. The owners provided a written informed consent prior to the participation. The horses were fed an unstandardized hay/haylage-based diet. Information on the use of specific FA supplements was not available. Inclusion criteria were as follows: no signs of infection during the last two months and no medication during the last month. Exclusion criteria included abnormalities in physical examinations (purulent nasal discharge, enlarged submandibular lymph nodes, fever, diarrhea, poor body condition), abnormal blood analyses (blood leukocyte or serum amyloid A concentration outside reference ranges), and abnormalities in airway endoscopy (laryngeal dysfunction, arytenoid chondropathy, pneumonia, neoplasia). Horses were diagnosed as asthmatic based on patient history (recurrent or chronic signs of sterile inflammatory lung disease) and neutrophilia (neutrophil-% of 5–25% for MMEA and > 25% for SEA) in BALF cytology. Horses were diagnosed as healthy based on patient history (no signs of recurrent or chronic lung disease in known history) and lack of neutrophilia (neutrophil-% of < 5%) in BALF cytology^25^. Only horses with neutrophilic form of asthma were included in the study^26^.

Arterial blood sampling, airway endoscopy, collection of BALF, and cytology were performed as described previously using detomidine (0.01 mg/kg, Domosedan, Orion, Espoo, Finland) and butorphanol (0.005–0.01 mg/kg, Butordol, Intervet, Boxmeer, the Netherlands) for sedation and 1% lidocaine solution (20–40 mL, Lidocain, Orion) for local anesthesia^27^. Collection of BALF was performed with sterile 0.9% saline by injecting 360 mL to horses and 240 mL to ponies in one aliquot, followed by manual aspiration of the fluid. The volume-% (mean ± standard error [SE]) retrieved were as follows: 54% (± 2.3) for control horses, 55% (± 3.6) for MMEA horses, and 48% (± 5.8) for SEA horses. The sample types prepared for FA analyses were BALF, supernatant (SUP), and EVs. For SUP, BALF was centrifuged at +4°C, 300 × *g* for 10 min followed by centrifugation of the supernatant at 3000×*g* for 20 min. The EV population (particle size up to 1000 nm) was isolated and purified with size-exclusion chromatography (SEC), as described previously^27^. One control horse did not have a SUP sample, and therefore the sample sizes were 30 BALF samples, 29 SUP samples, and 30 EV samples.

### FA determination

FA analysis of BALF, SUP, and EVs was performed at the Helsinki University Lipidomics Unit using gas chromatography with flame ionization and mass spectrometric detection as outlined previously^28^. FAs were released from lipids and converted to FA methyl esters (FAMEs) in a transmethylation reaction in methanolic H_2_SO_4_ under nitrogen atmosphere. The formed FAMEs were then extracted with hexane and analyzed by Shimadzu GC-2010 Plus gas chromatograph with flame ionization detector (Shimadzu, Kyoto, Japan). The FAME structures were verified with electron impact mass spectra recorded by the Shimadzu GCMS-QP2010 Ultra with a mass selective detector. The gas chromatographic peak representing docosahexaenoic acid (22:6n-3) also included an artefact, but this peak was regardless included in the analyses, as the artefact was not the major component of the peak, and it was estimated to be present with an equal proportion in all samples. The FA composition is presented as molar percentages (mol-%).

### Conventional statistics and discriminant analysis (DA)

Univariate statistical analyses and the DA were performed using the IBM SPSS *v*27 software (IBM, Armonk, NY, USA). To test for differences between the diagnosis groups, the Kruskal–Wallis test was used for body weight, trachea mucus score, arterial oxygen content, and cytology, the Fisherʼs exact test for sex distribution, and the one-way analysis of variance for age. Comparisons of FA composition between the diagnosis groups and sample types were performed with the generalized linear model, in which the variables showing significance were compared post hoc all groups pairwise. The Studentʼs t-test was used for comparisons of FA profiles between controls and pooled data of all asthmatic horses in different sample types. Correlations were calculated with the Spearman correlation coefficient (r_s_). The *p* < 0.05 was considered statistically significant and results are presented as the mean ± SE. In addition to univariate analyses, a range of multivariate analyses (see below) using both sample type-specific and combined datasets were performed to explore the data from complementary, non-overlapping angles. Those analyses were performed using FA composition data, and it is indicated below when individual FA proportions were supplemented with FA sums and derived indices, which were calculated as described previously^28^. The supervised DA was used to assess whether the diagnosis groups differed in the whole FA profile and which sample type best classified the samples into their respective groups. For cross-validation, we performed a leave-one-out strategy, to get insight on whether the obtained discriminant functions could correctly classify uncategorized samples^29^.

### Soft independent modeling of class analogy (SIMCA)

We used Principal Component Analysis (PCA, an unsupervised multivariate method) followed by SIMCA^30^ to test quantitatively, comparing two diagnosis groups at a time, whether the diagnosis groups had statistically different FA compositions. Arcsin-transformed and standardized FA mol-% data, i.e., with homogenized variable deviations, were used. The analyses were performed with the Sirius *v*8.5 software (Pattern Recognition Systems, Bergen, Norway).

### Hierarchical clustering (HC) and correlation analysis

For each FA or derived index when indicated, the measured abundance was converted to a Z-score (normalization across samples) using the IBM SPSS. Z-score data were then loaded onto ClustVis (https://biit.cs.ut.ee/clustvis/) and HC was performed using “correlations” as the clustering distance and the Ward clustering method^31^ for both rows (FA Z-scores) and columns (samples). Sample type and diagnosis were not used for clustering. Pearson (linear) correlation analysis was performed in R *v*4.1.2 (Team R 2020) using the corrplot library. FA clustering into 6 biologically relevant distinct groups was assessed visually from the correlogram and confirmed, in large part, from the (unsupervised) HC analysis.

### Random forest (RF) analysis

For RF analysis, a custom sklearn-based Python script was used, adapted from https://www.kaggle.com/code/prashant111/random-forest-classifier-tutorial/notebook, and provided as an annotated Jupyter notebook. The following parameters were used to build the RF classifier when using raw FA data (without enrichment, see below): n_estimators = 100, criterion = ‘entropy’, max_depth = 10, min_samples_split = 2, min_samples_leaf = 1, min_weight_fraction_leaf = 0.0, max_features = 8, max_leaf_nodes = None, min_impurity_decrease = 0.0, bootstrap = True, oob_score = False, n_jobs = -1, random_state = None, verbose = 0, warm_start = False, class_weight = None, ccp_alpha = 0.0, max_samples = None. Note that the maximal number of features to build each tree was adjusted to the dataset width (8 features for single FA data, 5 features for FA group-based data), in agreement with standard practices.

First, we probed the capacity of the RF model to recognize a sample with a known diagnosis based on different subsets of correlated FA variables. Since 6 groups of highly correlated FA variables (scores) were identified, the original dataset of 89 samples was enriched by creating reduced datasets of 6 features only, but with a 1000-fold number of samples obtained by randomly sampling one FA score from each group only once to create samples with 6 features for classification instead of including the scores of each FA. The dataset was then split into training and testing data by randomly selecting 80% of the enriched samples into a training set and 20% into a testing set. Second, we probed the capacity of the model to predict diagnosis of samples without including supervising information on diagnoses of the training samples. In this approach, the original data were split before enrichment into the training and testing sets and only then we performed the 1000-fold enrichment separately on these sets. The relevance of individual FAs or FA groups in estimating the diagnosis was quantified using sklearn’s feature_importances_function, and estimated from the training data only. We note that the prediction accuracy of the model, based on mean decrease in impurity, remained imperfect in the current study (see Results). Hence, it is possible that relevant FAs or FA groups were underestimated in the analysis. The RF prediction accuracy scores were defined based on averages of 20 leave-one-out RF runs.

### Ethics statement

The animal study was reviewed and approved by the Project Authorization Board in the Regional State Administrative Agency (ESAVI/3285/2020). Written informed consent was obtained from the owners for the participation of their animals in this study.

## Results

### Clinical variables

The general characteristics of the horses are described in Table 1. There were no differences in the average age, body weight, sex ratio, or arterial oxygen content between the groups, while the mucus score was significantly elevated in MMEA compared to control. Prevalent symptoms in EA horses reported by the owners included cough (n = 13), poor performance (n = 7), and abdominal breathing pattern (n = 9). Out of 15 horses with EA, 10 horses were diagnosed with MMEA (BALF neutrophils 11.3 ± 1.47%), and 5 horses with SEA (39.1 ± 5.76%). Typical clinical findings in EA horses were increased respiratory rate (n = 10), abdominal breathing pattern (n = 9), and abnormal respiratory auscultation (n = 8). Control horses had no abnormal clinical findings and the mean BALF neutrophil-% was 1.9 ± 0.24%.

**Table 1:** Characteristics of control horses and horses with mild/moderate asthma (MMEA) and severe asthma (SEA) (mean ± SE).

	Control (*n* = 15)	MMEA (*n* = 10)	SEA (*n* = 5)	*p*
Age (years)	12 ± 1	16 ± 1	12 ± 1	0.051
Body weight (kg)	524 ± 25	439 ± 43	517 ± 93	0.181
Sex				0.103
Mare (n/%)	3 (20%)	0 (0%)	2 (40%)	
Stallion (n/%)	0 (0%)	2 (20%)	0 (0%)	
Gelding (n/%)	12 (80%)	8 (80%)	3 (60%)	
Mucus score (1–5)	1.4 ± 0.19^a^	2.6 ± 0.31^b^	2.4 ± 0.93^ab^	0.028
PaO_2_ (mmHg)	102.0 ± 4.07	97.6 ± 3.52	87.2 ± 3.43	0.063
BALF				
Neutrophils (%)	1.9 ± 0.24^a^	11.3 ± 1.47^b^	39.1 ± 5.76^b^	< 0.001
Mast cells (%)	1.8 ± 0.34	2.3 ± 0.57	1.0 ± 0.22	0.550
Eosinophils (%)	0.1 ± 0.04	0.8 ± 0.40	0.2 ± 0.10	0.214
Lymphocytes (%)	47.8 ± 2.06^a^	55.4 ± 2.46^b^	40.3 ± 5.81^a^	0.041
Macrophages (%)	48.2 ± 2.29^b^	29.8 ± 2.65^a^	19.0 ± 2.01^a^	< 0.001

*PaO*_*2*_ arterial oxygen content, *BALF* bronchoalveolar lavage fluid.

Mucus score by Gerber et al.^32^. Different superscript letters indicate significant differences between the means within a row (Kruskal–Wallis test was used for body weight, trachea mucus score, PaO_2_, and cytology, the Fisherʼs exact test for sex distribution, and the one-way analysis of variance for age, *p* < 0.05).

### Comparison of FA profiles between sample types

**Figure 1:**
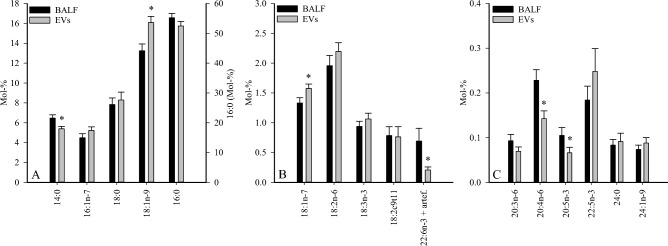
Comparison of proportions (mol-%) of selected fatty acids in bronchoalveolar lavage fluid (BALF) and its extracellular vesicles (EVs) in healthy and asthmatic horses (mean + SE). *Significant difference between sample types (generalized linear model, *p* < 0.05).

The detailed FA profiles of the samples are presented in Supplementary Tables 1–3. Compared to BALF, SUP contained higher proportions of total monounsaturated FAs (MUFAs), including *cis*-hypogeic acid (16:1n-9), *cis*-9-palmitoleic acid (16:1n-7), oleic acid (18:1n-9), and *cis*-vaccenic acid (18:1n-7), and lower percentages of total saturated FAs (SFAs), including myristic acid (14:0), nonadecylic acid (19:0), arachidic acid (20:0), and behenic acid (22:0). In addition, the proportions of essential C18 PUFAs (linoleic acid 18:2n-6 and *α*-linolenic acid 18:3n-3), unsaturated FA (UFA)/SFA ratios, ∆9-desaturation indices (DIs), and ∆5-DIs were higher and C20 n-6 PUFAs, eicosapentaenoic acid (20:5n-3), docosapentaenoic acid (22:5n-3), and product/precursor ratios of n-6 PUFAs were lower in SUP than in BALF. In the EV fraction, the proportions of 14:0, 20:5n-3, arachidonic acid (20:4n-6), and 22:6n-3 (incl. unidentified artefact) were lower together with lower product/precursor ratios of n-3 PUFAs compared to BALF (Fig. 1, Supplementary Tables 1, 3). In contrast, 18:1n-9 and 18:1n-7 showed elevated percentages in EVs.

### Comparison of FA profiles between diagnosis groups

#### BALF

**Figure 2:**
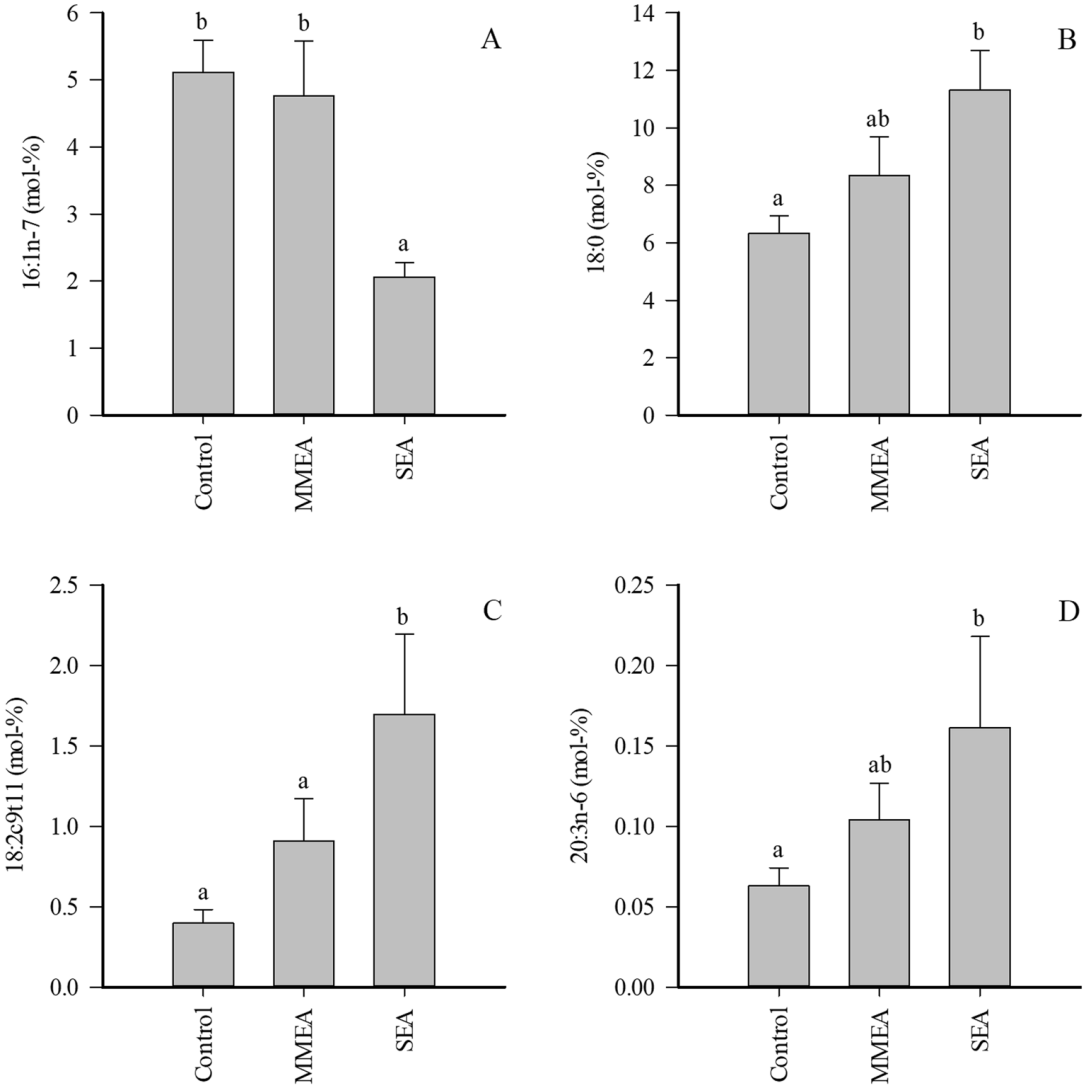
Proportions (mol-%) of selected fatty acids in bronchoalveolar lavage fluid of control horses and horses with mild/moderate asthma (MMEA) or severe asthma (SEA) (mean + SE). Dissimilar letters indicate statistically significant differences between diagnoses (generalized linear model, *p* < 0.05).

Regarding individual FAs with potential implications on lung physiology, the proportions of 16:1n-7 were lower and those of stearic acid (18:0), rumenic acid (18:2*c*9*t*11), and dihomo-*γ*-linolenic acid (20:3n-6) were higher in SEA than in healthy controls (Fig. 2). In addition, the proportions of margaric acid (17:0), anteisoheptadecanoic acid (17:0*ai*), 20:0, gadoleic acid (20:1n-11), paullinic acid (20:1n-7), and dihomolinoleic acid (20:2n-6) increased in SEA, and n-3/n-6 PUFA ratios and product/precursor ratios of both n-3 and n-6 PUFAs were elevated (Supplementary Table 1). In contrast, 16:1n-9 and ∆5-DIs were lower in SEA. The only significant difference in MMEA was the decreased proportion of 17:0*ai* compared to controls. When all asthmatic horses were grouped together and compared to controls, many of the above-mentioned differences remained significant (data not shown). In addition, 22:5n-3 increased in proportion in EA (0.119 ± 0.022 *vs.* 0.249 ± 0.054 mol-% for controls and all EA horses, respectively, Studentʼs t-test, *p* = 0.041).

There was a negative correlation between the percentages of essential PUFAs, 18:2n-6 and 18:3n-3, with BALF neutrophil-% (r_s_ =  − 0.443 to − 0.488, *p* = 0.006–0.014). The correlation was positive between BALF neutrophil-% and the proportions of 18:0 and 18:2*c*9*t*11 (r_s_ = 0.472–0.518, *p* = 0.003–0.009), while the correlation was negative for 16:1n-7 (r_s_ =  − 0.459, *p* = 0.011). BALF macrophage-% correlated positively with 16:1n-7, 18:2n-6, and 18:3n-3 (r_s_ = 0.466–0.553, *p* = 0.002–0.009) and negatively with 18:0, 18:2*c*9*t*11, 20:3n-6, 20:4n-6, and lignoceric acid (24:0) (r_s_ =  − 0.403 to − 0.529, *p* = 0.003–0.027).

#### SUP

**Figure 3:**
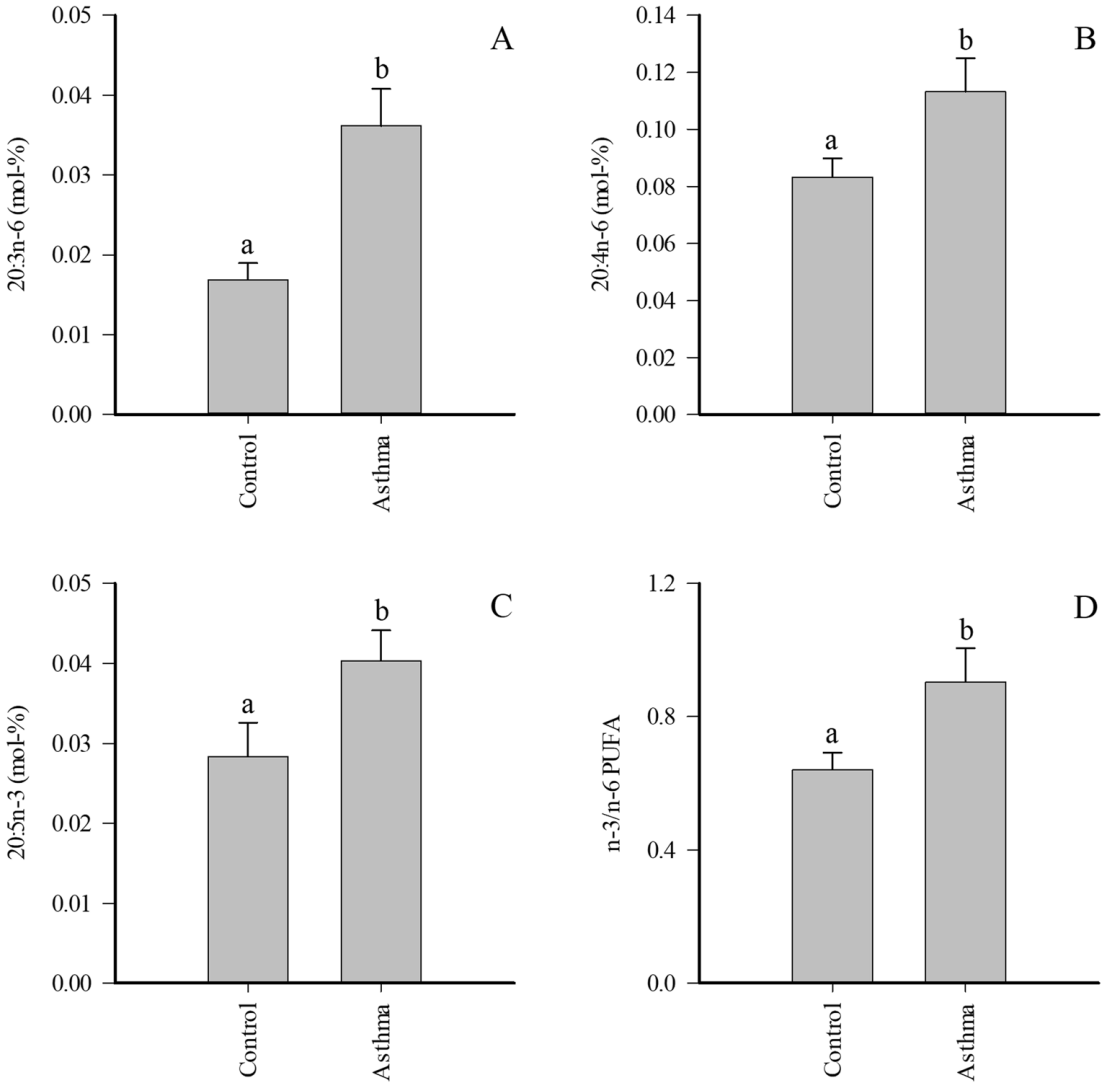
Proportions (mol-%) of selected fatty acids in supernatant of bronchoalveolar lavage fluid of control horses and pooled asthmatic horses (mean + SE). Dissimilar letters indicate statistically significant differences between diagnoses (generalized linear model, *p* < 0.05). *PUFA* polyunsaturated fatty acid.

MMEA was characterized with elevated proportions of several FAs with minor proportions when compared to controls, including 20:0, 20:3n-6, 22:5n-3, and 24:0, while the differences between control and SEA did not reach significance (Supplementary Table 2). ∆5-DIs were reduced and product/precursor ratios of n-6 PUFAs elevated in MMEA. When the pooled asthma samples were compared to controls, most of the above-mentioned differences remained significant and 20:4n-6, 20:5n-3, and n-3/n-6 PUFA ratios also increased in EA (Fig. 3).

There was a positive correlation between BALF neutrophil-% and the proportion of 18:2*c*9*t*11, 20:3n-6, 20:5n-3, and 24:0 (r_s_ = 0.393–0.602, *p* = 0.001–0.035). 20:3n-6 correlated negatively with BALF macrophage-% (r_s_ =  − 0.437, *p* = 0.018), and 20:4n-6 with BALF mast cell-% (r_s_ = –0.400, *p* = 0.032).

#### EVs

**Figure 4:**
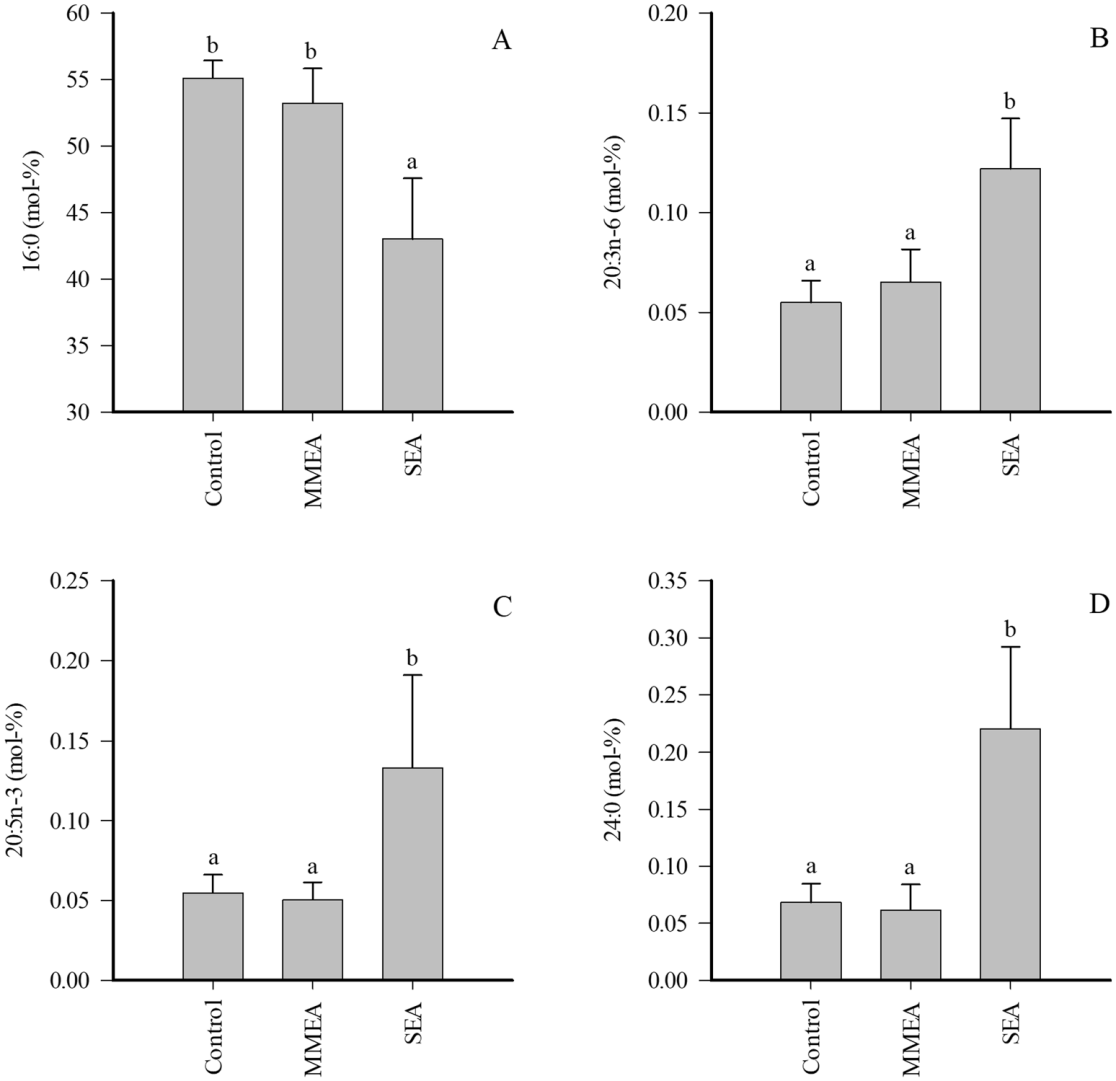
Proportions (mol-%) of selected fatty acids in extracellular vesicles of bronchoalveolar lavage fluid of control horses and horses with mild/moderate asthma (MMEA) or severe asthma (SEA) (mean + SE). Dissimilar letters indicate statistically significant differences between diagnoses (generalized linear model, *p* < 0.05).

The EV proportions of 16:0 markedly decreased in SEA (Fig. 4), and this was accompanied with a reduction in total SFAs and increased UFA/SFA ratios, double bond indices, and ∆9-DIs (Supplementary Table 3). In addition, the percentages of 20:3n-6, 20:5n-3, 24:0 (Fig. 4), and several minor 14–20C SFAs and MUFAs increased in SEA together with elevated product/precursor ratios of n-6 PUFAs. All statistically significant differences except for one (20:0) disappeared with pooled EA samples.

The EV proportion of 18:2n-6 was positively correlated with BALF macrophage-% (r_s_ = 0.422, *p* = 0.020) and negatively with BALF neutrophil-% (r_s_ =  − 0.418, *p* = 0.021). 16:0 correlated positively with BALF mast cell-% (r_s_ = 0.375, *p* = 0.041). There was a negative correlation between 20:4n-6 and BALF macrophage-% (r_s_ =  − 0.362, *p* = 0.049), while 20:3n-6 correlated positively with BALF neutrophil-% (r_s_ = 0.378, *p* = 0.040) and negatively with BALF macrophage-% (r_s_ =  − 0.411, *p* = 0.024).

### Discriminant analysis

In order to see if the FA differences were sufficient to distinguish sample types, we performed DA where the BALF FA profiles were separated from the corresponding SUP and EVs, which aligned closer to each other (Supplementary Fig. 1). The analysis classified 95.5% of samples correctly into their respective groups. The FAs with the largest separation power included 16:1n-9 (function 1), 20:1n-11, 20:1n-7, gondoic acid (20:1n-9), and 18:1n-9 (function 2).

**Figure 5:**
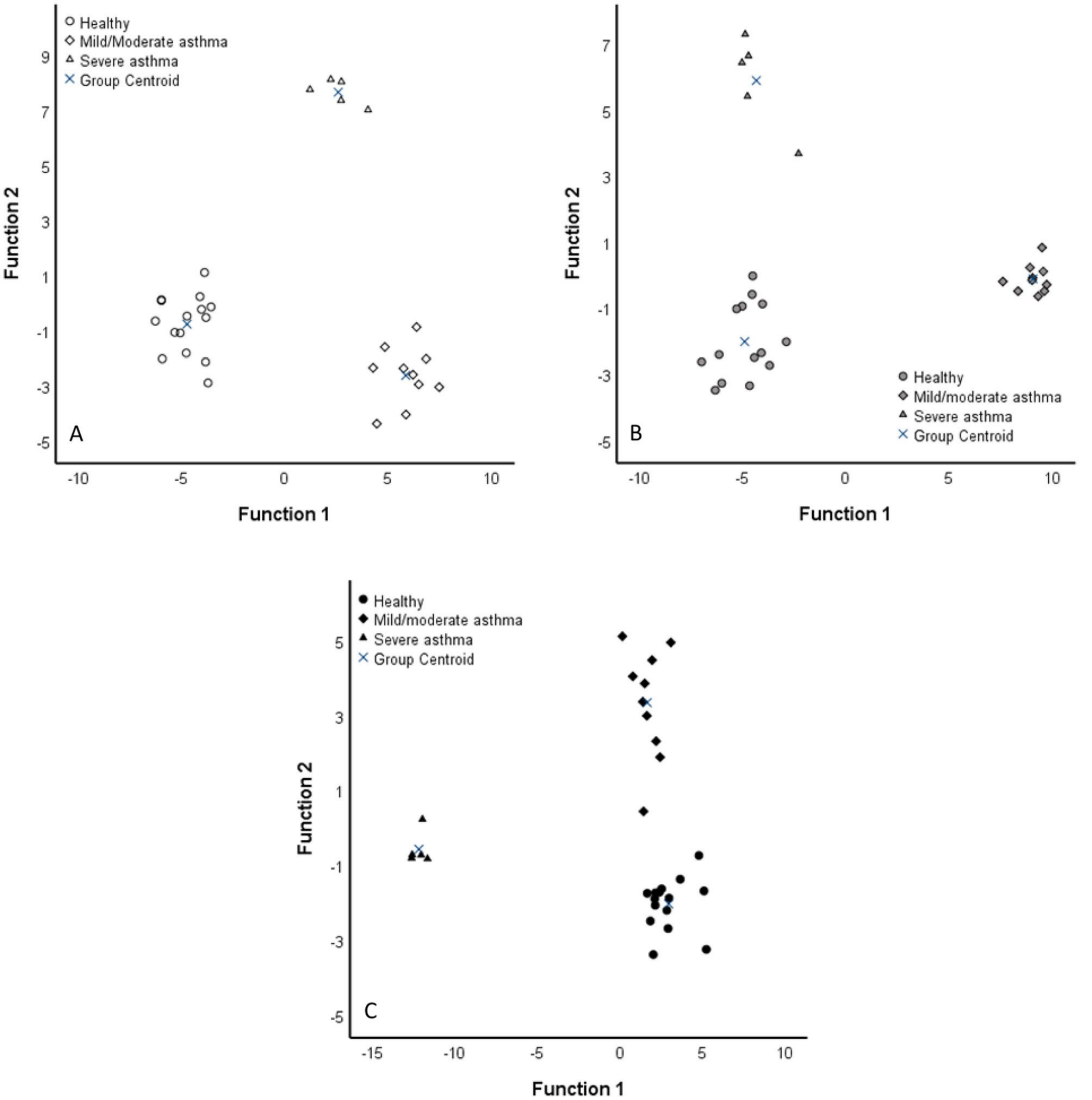
Discriminant analyses depicting the classification of fatty acid data in equine bronchoalveolar lavage fluid (BALF, **A**), its supernatant (SUP, **B**), and extracellular vesicles (EVs, **C**) of control horses and horses with mild/moderate asthma or severe asthma based on discriminant functions 1 (x-axis) and 2 (y-axis). White symbols = BALF, grey symbols = SUP, black symbols = EVs. See “Results” for details.

In order to test which sample type provides the best separation power in FA analysis, the DA was repeated on BALF, SUP, and EV samples separately, with the diagnosis as the grouping variable (Fig. 5). In BALF, the analysis classified 100% of the samples into their correct diagnosis group. The variables with the largest separation power included palmitvaccenic acid (16:1n-5), pentadecylic acid (15:0), 18:1n-7, and isopentadecylic acid (15:0*i*). In SUP, the analysis also classified the samples into correct diagnosis groups with 100% accuracy. The most important contributors to the model were anteisopentadecylic acid (15:0*ai*), myristoleic acid (14:1n-5), 14:0, and 18:1n-9. In EVs, the analysis classified 96.7% of samples correctly into diagnosis groups. The FAs with the largest separation power included 16:1n-5, 15:0*ai*, 24:0, and isomyristic acid (14:0*i*). Although all these DAs classified most (> 95%) of the samples into their correct diagnosis group, only 50–60% accuracy (depending on sample type) was reached when performing leave-one-out cross-validation.

### Soft independent modeling of class analogy

**Figure 6:**
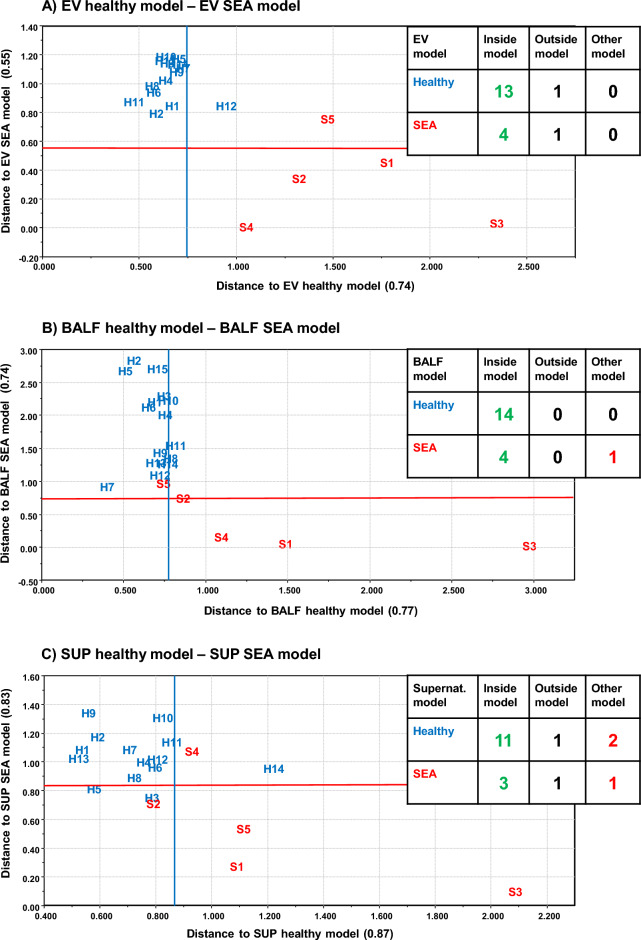
Principal component analysis model-based soft independent modeling of class analogies classification of extracellular vesicles (EV, **A**), bronchoalveolar lavage fluid (BALF, **B**), and supernatant (SUP, **C**) from healthy and severely asthmatic (SEA) horses by using standardized fatty acid mol-% data and drawing model boundaries with *p* < 0.05 level. Number of samples fitting inside own model, outside own model, and inside other model are shown in table inserts. Accurate sample location is in the lower left corner of the sample code (*H* healthy horse, *S* SEA horse with numbers of individuals).

SIMCA (using group models based on principal component analysis PCA) separated the EV FA profiles of SEA horses from controls (*p* < 0.05) (Fig. 6) but did not show separation between SEA and MMEA. In the PCA, the FAs 16:0 and 20:3n-6 had large separation power and correlated negatively with each other. 16:0 was enriched in controls and 20:3n-6 in SEA horses. The SEA *vs.* control comparisons using the BALF and SUP samples did not reach statistical significance, despite the majority of samples located inside their own PCA-based models. The BALF, SUP, and EV samples from MMEA horses did not differ from healthy nor SEA samples (data not shown).

### Hierarchical clustering and correlation analysis

**Figure 7:**
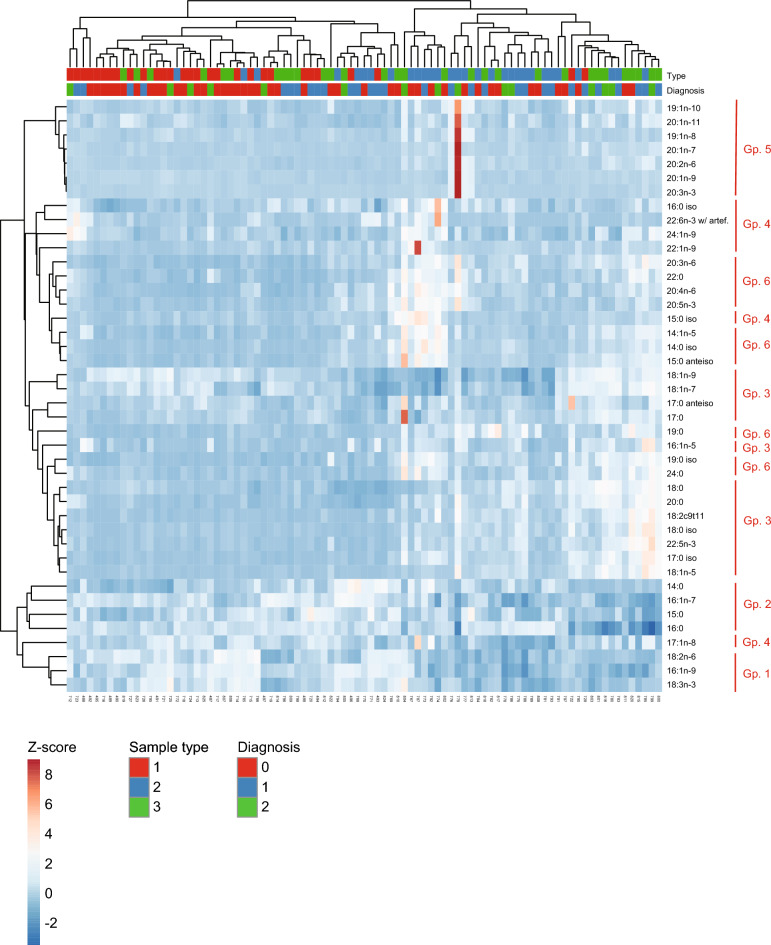
Hierarchical clustering identifies groups of fatty acids (FAs) with consistent profiles across diagnostic groups. Clustergram showing the FA Z-scores (rows) in samples (columns), color-coded as indicated. Hierarchical clustering of the FAs (bonds on the left of the clustergram; distance of bond to color-coded clustergram grows with the dissimilarity between FAs across samples), and of the samples (bonds at the top of the clustergram; distance of bond to color-coded clustergram grows with the dissimilarity between samples across FA space) was performed in ClustVis (https://biit.cs.ut.ee/clustvis/) using the Ward method^31^. The type of sample (1 = supernatant; 2 = bronchoalveolar lavage fluid; 3 = extracellular vesicles), and the inflammatory state of the corresponding horse (0 = healthy, 1 = mild/moderate asthma, 2 = severe asthma) are indicated on top of the clustergram. Groups (Gp.) of FAs that cluster samples are indicated with red vertical bars on the right.

The correlations described above between FA abundances and BALF neutrophil-% in all sample types suggested that several FAs themselves might show strong correlations across samples. To test this hypothesis, all FA–FA Pearson’s correlations (r_p_) were computed across samples. These data (Supplementary Table 4, Supplementary Fig. 2) revealed 6 distinct FA groups with strong and statistically significant intra-group correlations (r_p_ > 0.4, often > 0.7–0.8). The same FA group structure was confirmed to a very large extent using unsupervised HC (Fig. 7). Further, the FA groups were biologically relevant with, for instance, group 2 being mostly formed of C14–16 SFAs. Clustering of the sample types or the diagnoses of the horses was only partially achieved (Fig. 7, top rows), in agreement with DA cross-validation. This suggested that the FA profiles from our 89 samples were only partially capable of predicting diagnosis for uncategorized samples.

### Random forest tree analyses of FA profiles

To estimate the predicting power of FA profiles, a RF analysis was performed. In RF-based classification of the samples’ inflammatory state (as diagnosed), the 6 FA groups got different importance scores (Supplementary Fig. 3), and as a positive control, RF perfectly predicted samples’ diagnosis when based on immune cells-%, predominantly neutrophil- and macrophage-% (Supplementary Fig. 4). When selecting randomly the samples from the enriched dataset into training and testing sets, the approach yielded 99.99% prediction accuracy on the test data, demonstrating that the subsets of the full FA dataset included for training were sufficient to predict other FA subsets from the same samples, preserving the information from the full dataset. This result emphasized the relevance of the FA groups, as it demonstrated that they contained redundant information.

When the grouped FA data were enriched after splitting into training and testing samples, the prediction accuracy was 40–59% depending on sample type and whether derived variables were excluded or included (Supplementary Table 5). FA groups 1 (16:1n-9, 18:2n-6, and 18:3n-3) and 2 (14:0, 15:0, 16:0, and 16:1n-7) were most frequently relevant to the RF model outcomes when all sample types were analyzed together (Supplementary Table 4, Supplementary Fig. 3), or samples were taken from EVs only (data not shown). However, when restricted to BALF samples, the most important features were Groups 1 and 6 (that includes 20:3n-6). These differences in the importance for diagnosis classification of different FA groups were even more apparent when the abundance of individual FAs was used for classification (Supplementary Figs. 5, 6), with 20:3n-6 as the topmost feature for BALF samples, in agreement with other analyses of this study.

## Discussion

This study investigated the pathophysiology of EA by describing the FA profiles of equine BALF and its components, and FA alterations that reflect the airway inflammatory status of asthmatic horses. This study demonstrates differences in FA profiles of different BALF components and between horses with EA and healthy horses. The potential role of BALF EVs as carriers of FAs with impact on the progression and resolution of inflammation and airway dysfunction is supported by the results. The differences in FA profiles between EA and healthy horses are not sufficient for diagnostics tools, however, the FA features reflect the inflammatory status of the animals. The horses in this study had naturally occurring disease, which improves the quality of this translational animal model and applicability of the results to human patients.

The enrolled horses in our study epitomized a natural horse population where MMEA is more prevalent compared to SEA. However, significant overlap in terms of EA severity-based subcategories exists and a clear-cut difference cannot be expected in the biochemical markers of inflammation. The clinical status of EA horses also affects the lipid composition of the airways, as shown by Christmann et al*.*^33^, where cyclic phosphatidic acid 16:0 and diacylglycerol 36:2 were elevated in the surfactant of EA horses during induced disease exacerbation. There is still no consensus if MMEA and SEA represent two distinct diseases or if they belong to the same continuum^2^. In this study, the FA profiles of three different sample types were analyzed. BALF contains cells and molecules, such as proteins and lipids, while SUP represents cell-free BALF, and the EVs contain the SEC-purified EV fraction. The MMEA and SEA samples showed distinct FA compositions when compared to healthy horses. Only three FAs (20:0, 20:1n-7, and 20:3n-6) showed elevated proportions in asthma samples in all three sample types. Thus, mainly different individual FAs were responsible for the discrimination of the diagnoses in BALF, SUP, and EVs. These distinctive FA profiles indicate differences in FA transfer from cells into the surrounding fluid and EVs, and that EVs might serve as a targeted delivery system and carry FAs that potentially modulate immune functions.

The supervised DA yielded good separation power when the diagnosis of each sample was known. In the absence of any a priori knowledge, a PCA-based SIMCA and leave-one-out DA were also performed. With these unsupervised analyses, the clearest separation of healthy and SEA horses was in the EV fraction. The ability of RF classification to predict the diagnosis based on FA profiles was approximately 60% at its best. This limited performance might originate from the small number of animals in the study, and it would be informative to repeat similar approaches on larger datasets. However, as explained above, asthma is not a disease that can always be diagnosed in a clear-cut manner, and therefore the 60% classification of the unsupervised methods was not unexpected. Moreover, the main aim of the study was not to diagnose asthma with FA profiles, but to assess the significance of FAs in disease pathogenesis and prediction.

Surfactant facilitates lung function by reducing tension and preventing the alveoli and small airways from collapsing, hence the impaired activity of surfactant complicates the function of the airways^34^. Previous research as far as from three decades ago revealed altered surfactant aggregate and protein composition as well as surfactant dysfunction in humans with allergen challenge^35^. Phosphatidylcholine 16:0/16:0 is the main phospholipid species of surfactant^36^. Christmann et al*.*^37^ found lower concentrations of surfactant phospholipids in cell-free BALF and surfactant pellet in EA horses in remission and exacerbation compared to control horses. In our study, the decreased 16:0 and total SFAs in EVs in SEA suggest that EVs could have a role in the transfer of FAs crucial to surfactant function. However, another study on EA horses observed that cell-free surfactant pellets of SEA individuals had increased levels of sphingolipids with 16:0 acyl chain and cyclic phosphatidic acid 16:0^23^.

Our results revealed an increase in BALF 18:0 mol-% in SEA horses. Evidence of inflammatory actions of SFAs has emerged in human asthma, where lysophosphatidylcholine 18:0 and 16:0 were elevated in BALF of patients with lung function impairment characteristic of asthma^38^. On the other hand, increased SFAs, such as 18:0, might also indicate the presence of plasma lipoproteins in BALF, caused by leakage from capillaries to alveoli in asthma^34^. In general, the BALF, SUP, and EVs of asthmatic horses contained elevated relative amounts of C20–24 SFAs, characteristic to lung sphingolipids^39^. Also Sánchez-Rodríguez et al*.*^40^ found an increase in SFA 22:0 in erythrocytes of human asthma patients, and reported that 24:0 could be a useful biomarker of lung cancer. Since induction of membrane rafts rich in sphingolipids (ceramide, sphingomyelin, and complex glycosphingolipids) has been associated with vesicle budding^41^, asthma may modulate vesiculation in the horse lung. Due to vesicle interleaflet coupling by acyl chain digitation, an increase in outer leaflet sphingolipids having C20–24 acyl chains also necessitates an elevation in inner leaflet phosphatidylserine 18:0/18:1, thus, also increasing the need for C18 FAs^42^. In our study, the EVs were enriched with 18:1n-9, resembling the findings from earlier studies where MUFAs, especially 18:1, were elevated in the FA profile of EVs from human prostate cancer cells^43^ and fibroblast-like synoviocytes^44^. Hough et al.^1^ also documented increased sphingomyelin species 34:1 (consisting of a 18:1 sphingoid base and 16:0 acyl chain) in BALF EVs of smoke-exposed asthmatic patients.

In the present study, 20:4n-6, which regulates immune responses in hypersensitivity reactions and serves as a precursor for eicosanoids^45^, increased in the SUP samples of pooled EA horses. Eicosanoids from 20:4n-6, such as prostaglandin E_2_ (PGE_2_) and cysteinyl leukotrienes, are bioactive lipid mediators primarily associated with inflammatory conditions and pain^46^. In the respiratory system, however, PGE_2_ has beneficial effects, as it increases relaxation of airway smooth muscle and inhibits the release of mast cell mediators and the recruitment of inflammatory cells^13^. Cysteinyl leukotrienes, on the other hand, play a consequential role in human asthma as potent bronchoconstrictors^47^. Derivatives of 20:4n-6 also include prostaglandins and lipoxins (early resolution SPMs) that can have pro-resolving actions and thereby attenuate inflammatory responses, impede tissue remodeling, and inhibit bronchoconstriction^13,48,49^. The function of 20:4n-6 is complex, however, in our study its increase in the SUP of asthmatic horses could be linked to the chronic inflammation present in the airways.

We observed an inverse association between 18:2n-6 and neutrophil-% in BALF and EV samples, and a similar phenomenon was documented by Sánchez-Rodríguez et al*.*^40^, who reported that erythrocyte 18:2n-6 decreased in patients with lung adenocarcinoma and squamous cell lung carcinoma. The other essential PUFA, 18:3n-3, also showed a negative association with BALF inflammation. A potential reason for the inflammation-related decrease in the essential PUFAs could be their intensified elongation and desaturation to lipid mediator precursors^50^, such as 20:3n-6 and 20:5n-3, that increased in SEA EVs. In addition to alterations in pro-inflammatory PUFAs, also those with known anti-inflammatory actions, such as 20:5n-3 and conjugated 18:2*c*9,*t*11^51^, increased in proportion in EV samples and/or BALF by EA. The increase in n-3 PUFAs may be beneficial in the resolving phase of inflammation, as cells can convert them to SPMs, such as resolvins, protectins, and maresins, in order to restore the homeostasis in the airways during inflammation^14^.

The proportions of 20:3n-6 increased in all sample types in EA. Woods et al*.*^52^ identified a positive association in young adults’ plasma 20:3n-6 levels and asthma, and increased proportions were noticed in the synovial membrane of patients with rheumatoid arthritis^53^, suggesting a crucial role for 20:3n-6 in chronic inflammatory conditions. 20:3n-6 is also the precursor of the anti-inflammatory prostaglandin E_1_ (PGE_1_), which promotes vasodilation, lowers blood pressure, and relaxes smooth muscle and, thereby, has the potential to improve lung function^54,55^. An in vitro study by Martin et al*.*^56^ demonstrated anti-inflammatory effects of the PGE_1_ analog, misoprostol, on equine blood neutrophils, mediated through inhibited neutrophil adhesion and migration in a dose-dependent manner. Our FA results suggest that, in addition to sustaining PUFA-mediated inflammatory processes, asthmatic lungs could simultaneously induce compensatory mechanisms to ameliorate inflammation and bronchoconstriction.

The present results show that, in horses with naturally occurring asthma, airway inflammation is associated with altered FA profiles in all examined BALF components, which indicates that the asthmatic airways react to the situation by altered FA proportions with both pro- and anti-inflammatory effects. Although the different sample types showed unique responses of FA composition to asthma, the elevated 20:3n-6 was an asthma marker common to all sample types. In SEA horses, EVs also had increased proportions of the anti-inflammatory 20:5n-3, and decreased proportions of the main surfactant FA, 16:0. The supervised DA method separated samples based on diagnoses excellently, while the ability of RF classification to predict the diagnosis based on FA profiles remained insufficient for diagnostics. Based on PCA–SIMCA, however, the FA profile of EVs was distinctly different between healthy and SEA horses indicating EVs as potential mediators in asthma pathogenesis. In this study, the SFA and PUFA components of EVs that responded to asthma are known to play central roles in inflammatory signaling pathways. Thus, the FA results provide new insight to the development and possible treatment of EA. Future studies should investigate the EV FA profiles in different inflammatory phases, in order to explore the potential of EVs as carriers of particular anti-inflammatory FAs as a part of asthma therapy.

### Study limitations

This study focused on FAs instead of neutral or phospholipids, therefore, the lipid class profiles of EV membranes in naturally occurring EA remain to be investigated. Blood contamination of BALF (a potential source of increased lipoprotein concentration) is possible but unlikely in this study, as no red blood cells were detected in the samples. In addition, SEC as a purification method for EVs may not remove all lipoprotein and protein residues from the samples, which can affect the EV FA profiles^57^. Finally, the individual diets of the horses could have caused variation in FA profiles, and the limited number of animals could have reduced the predicting value of FAs.

## Supplementary Information

Supplementary Information.

## Data Availability

The original contributions presented in the study are included in this article and its Supplementary Materials, further inquiries can be directed to the corresponding author.

## Abbreviations

BALF: Bronchoalveolar lavage fluid
DA: Discriminant analysis
DI: Desaturation index
EA: Equine asthma
EV: Extracellular vesicle
FA: Fatty acid
FAME: Fatty acid methyl ester
HC: Hierarchical clustering
MMEA: Mild/moderate equine asthma
MUFA: Monounsaturated fatty acid
PCA: Principal component analysis
PGE_1_: Prostaglandin E_1_
PGE_2_: Prostaglandin E_2_
PUFA: Polyunsaturated fatty acid
RF: Random forest
r_p_: Pearson correlation coefficient
r_s_: Spearman correlation coefficient
SE: Standard error
SEA: Severe equine asthma
SEC: Size-exclusion chromatography
SFA: Saturated fatty acid
SIMCA: Soft independent modeling of class analogies
SPM: Specialized pro-resolving mediator
SUP: Supernatant
UFA: Unsaturated fatty acid
