# Supplementary material for: Fatty acid fingerprints in bronchoalveolar lavage fluid and its extracellular vesicles reflect equine asthma severity
Source: Sci Rep. 2023 Jun 17;13:9821. doi: 10.1038/s41598-023-36697-x (PMC10276833; doi:10.1038/s41598-023-36697-x)
Supplement: Supplementary file 1 — Supplementary Information. [file 41598_2023_36697_MOESM1_ESM.pdf]

Supplementary table 1. Fatty acid (FA) proportions (mol-%, listed in the order of retention times of chromatographic peaks), sums, and indices in the bronchoalveolar lavage fluid of horses according to the severity of asthma (mean ± SE).

**FA/sum/index**	**Control**	**MMEA**	**SEA**	***P***
14:0*i*	0.299 ± 0.085	0.347 ± 0.153	0.538 ± 0.198	0.479
14:0	6.570 ± 0.443	6.765 ± 0.743	5.536 ± 0.531	0.433
14:1n-5	0.131 ± 0.034	0.172 ± 0.053	0.283 ± 0.098	0.148
15:0*i*	0.133 ± 0.037	0.104 ± 0.040	0.129 ± 0.052	0.851
15:0*ai*	0.182 ± 0.046	0.196 ± 0.046	0.262 ± 0.067	0.607
15:0	0.403 ± 0.028	0.357 ± 0.055	0.366 ± 0.058	0.659
16:0*i*	0.435 ± 0.040	0.392 ± 0.036	0.638 ± 0.216	0.098
16:0	57.107 ± 0.900	54.879 ± 3.190	50.248 ± 5.026	0.187
16:1n-9	0.382 ± 0.041[b]	0.298 ± 0.056[ab]	0.187 ± 0.022[a]	0.030
16:1n-7	5.116 ± 0.478[b]	4.765 ± 0.814[b]	2.059 ± 0.218[a]	0.007
16:1n-5	0.043 ± 0.007	0.073 ± 0.024	0.091 ± 0.018	0.095
17:0*i*	0.184 ± 0.030	0.298 ± 0.096	0.421 ± 0.104	0.061
17:0*ai*	0.196 ± 0.015[b]	0.146 ± 0.017[a]	0.236 ± 0.017[c]	0.003
17:0	0.341 ± 0.033[a]	0.351 ± 0.043[a]	0.507 ± 0.035[b]	0.021
17:1n-8	0.282 ± 0.046	0.211 ± 0.036	0.141 ± 0.024	0.116
18:0*i*	0.145 ± 0.020	0.286 ± 0.105	0.310 ± 0.087	0.127
18:0	6.327 ± 0.615[a]	8.342 ± 1.352[ab]	11.310 ± 1.396[b]	0.006
18:1n-9	13.195 ± 0.589	13.080 ± 1.417	13.730 ± 2.689	0.946
18:1n-7	1.268 ± 0.086	1.375 ± 0.194	1.439 ± 0.287	0.733
18:1n-5	0.131 ± 0.039	0.343 ± 0.152	0.492 ± 0.213	0.070
19:0*i*	0.063 ± 0.012	0.075 ± 0.015	0.081 ± 0.013	0.655
18:2n-6	2.276 ± 0.276	1.808 ± 0.244	1.299 ± 0.165	0.068
19:0	1.186 ± 0.337	1.507 ± 0.470	2.010 ± 0.578	0.456
19:1n-10	0.043 ± 0.008	0.048 ± 0.011	0.109 ± 0.070	0.118
19:1n-8	0.050 ± 0.014	0.050 ± 0.016	0.193 ± 0.154	0.094
18:3n-3	1.091 ± 0.128	0.888 ± 0.134	0.580 ± 0.148	0.063
18:2*c*9*t*11	0.397 ± 0.086[a]	0.909 ± 0.264[a]	1.696 ± 0.500[b]	0.000456
20:0	0.096 ± 0.011[a]	0.142 ± 0.034[a]	0.246 ± 0.039[b]	0.000369
20:1n-11	0.018 ± 0.003[a]	0.019 ± 0.005[a]	0.058 ± 0.031[b]	0.012
20:1n-9	0.157 ± 0.032	0.146 ± 0.042	0.689 ± 0.580	0.080
20:1n-7	0.009 ± 0.002[a]	0.013 ± 0.004[a]	0.076 ± 0.060[b]	0.023
20:2n-6	0.100 ± 0.020[a]	0.130 ± 0.030[a]	0.427 ± 0.302[b]	0.043
20:3n-6	0.063 ± 0.011[a]	0.104 ± 0.023[ab]	0.161 ± 0.057[b]	0.016
20:4n-6	0.221 ± 0.037	0.230 ± 0.035	0.247 ± 0.061	0.925
20:3n-3	0.130 ± 0.049	0.138 ± 0.057	1.007 ± 0.935	0.074
20:5n-3	0.092 ± 0.021	0.097 ± 0.022	0.159 ± 0.079	0.354
22:0	0.031 ± 0.007	0.031 ± 0.008	0.054 ± 0.020	0.270
22:1n-9	0.200 ± 0.091	0.094 ± 0.021	0.093 ± 0.021	0.494
22:5n-3	0.119 ± 0.022	0.253 ± 0.079	0.240 ± 0.050	0.074
24:0	0.076 ± 0.023	0.083 ± 0.016	0.105 ± 0.021	0.724
22:6n-3 (w/ artefact)	0.630 ± 0.175	0.404 ± 0.200	1.454 ± 1.148	0.210
24:1n-9	0.083 ± 0.015	0.052 ± 0.012	0.090 ± 0.025	0.222
Σ:SFA	73.773 ± 0.903	74.300 ± 2.329	72.999 ± 4.446	0.922
Σ:MUFA	21.107 ± 0.784	20.739 ± 1.871	19.731 ± 3.586	0.859
Σ:PUFA	5.120 ± 0.460	4.961 ± 0.641	7.270 ± 2.191	0.176
Σ:n-6 PUFA	2.660 ± 0.273	2.272 ± 0.266	2.134 ± 0.473	0.433
Σ:n-3 PUFA	2.062 ± 0.263	1.780 ± 0.330	3.439 ± 1.362	0.093
UFA/SFA	0.358 ± 0.016	0.358 ± 0.043	0.392 ± 0.089	0.832
n-3/n-6 PUFA (w/ 22:6n-3)	0.796 ± 0.092[a]	0.772 ± 0.092[a]	1.509 ± 0.524[b]	0.017
n-3/n-6 PUFA (w/o 22:6n-3)	0.560 ± 0.047	0.631 ± 0.078	0.736 ± 0.245	0.440
TACL	16.399 ± 0.031	16.443 ± 0.083	16.497 ± 0.100	0.571
Δ9-DI	0.289 ± 0.014	0.292 ± 0.035	0.279 ± 0.064	0.960
Δ5-DI (n-6 PUFA)	3.850 ± 0.389[b]	2.683 ± 0.349[ab]	1.832 ± 0.263[a]	0.002
DBI	0.362 ± 0.018	0.349 ± 0.036	0.435 ± 0.083	0.289
Prod/prec (n-6 PUFA)	0.151 ± 0.032[a]	0.213 ± 0.039[ab]	0.323 ± 0.081[b]	0.029
Prod/prec (n-3 PUFA)	0.686 ± 0.159[a]	0.467 ± 0.153[a]	4.735 ± 3.914[b]	0.033

MMEA = mild/moderate asthma, SEA = severe asthma, *i* = *iso*-methyl-branch, *ai* = *anteiso*-methyl-branch, *c* = *cis*, *t* = *trans*, Σ = sum, SFA = saturated FA, MUFA = monounsaturated FA, PUFA = polyunsaturated FA, UFA = unsaturated FA, TACL = total average chain length, Δ9-DI = delta-9 desaturation index, Δ5-DI = delta-5 desaturation index, DBI = double bond index, prod = product, prec = precursor, different superscript letters indicate significant differences between the means within a row (generalized linear model, $p < 0.05$)

Supplementary table 2. Fatty acid (FA) proportions (mol-%, listed in the order of retention times of chromatographic peaks), sums, and indices in the supernatant of bronchoalveolar lavage fluid of horses according to the severity of asthma (mean ± SE).

**FA/sum/index**	**Control**	**MMEA**	**SEA**	***P***
14:0*i*	0.042 ± 0.008^{a}	0.125 ± 0.023^{b}	0.090 ± 0.047^{ab}	0.002
14:0	4.698 ± 0.201	6.090 ± 0.817	4.830 ± 0.526	0.090
14:1n-5	0.054 ± 0.016^{a}	0.148 ± 0.039^{b}	0.089 ± 0.028^{ab}	0.021
15:0*i*	0.049 ± 0.005	0.064 ± 0.005	0.059 ± 0.003	0.070
15:0*ai*	0.051 ± 0.006^{a}	0.120 ± 0.018^{b}	0.079 ± 0.017^{ab}	0.000095
15:0	0.383 ± 0.032	0.435 ± 0.068	0.427 ± 0.031	0.667
16:0*i*	0.316 ± 0.028	0.339 ± 0.046	0.396 ± 0.046	0.403
16:0	56.108 ± 0.598	53.913 ± 1.883	55.306 ± 1.670	0.396
16:1n-9	0.457 ± 0.027	0.406 ± 0.042	0.521 ± 0.069	0.185
16:1n-7	6.113 ± 0.370	6.294 ± 0.484	6.059 ± 0.701	0.934
16:1n-5	0.038 ± 0.008^{a}	0.085 ± 0.021^{b}	0.048 ± 0.016^{ab}	0.033
17:0*i*	0.067 ± 0.005^{a}	0.154 ± 0.046^{b}	0.084 ± 0.012^{ab}	0.037
17:0*ai*	0.182 ± 0.011	0.229 ± 0.070	0.181 ± 0.018	0.638
17:0	0.294 ± 0.018	0.335 ± 0.038	0.339 ± 0.035	0.414
17:1n-8	0.278 ± 0.017	0.242 ± 0.023	0.311 ± 0.032	0.135
18:0*i*	0.081 ± 0.007	0.181 ± 0.063	0.106 ± 0.012	0.097
18:0	5.653 ± 0.190	6.875 ± 0.972	5.683 ± 0.129	0.218
18:1n-9	17.657 ± 0.445	15.578 ± 0.964	17.286 ± 1.592	0.107
18:1n-7	1.502 ± 0.059	1.561 ± 0.124	1.710 ± 0.083	0.344
18:1n-5	0.025 ± 0.003	0.099 ± 0.043	0.042 ± 0.006	0.061
19:0*i*	0.016 ± 0.001^{a}	0.040 ± 0.011^{b}	0.021 ± 0.003^{ab}	0.014
18:2n-6	2.940 ± 0.256	2.349 ± 0.190	2.535 ± 0.461	0.201
19:0	0.358 ± 0.054	0.813 ± 0.266	0.398 ± 0.063	0.067
19:1n-10	0.036 ± 0.003	0.052 ± 0.016	0.033 ± 0.006	0.386
19:1n-8	0.045 ± 0.004	0.045 ± 0.007	0.047 ± 0.010	0.972
18:3n-3	1.322 ± 0.125	1.093 ± 0.119	1.502 ± 0.441	0.324
18:2*c*9*t*11	0.106 ± 0.015	0.325 ± 0.131	0.161 ± 0.047	0.080
20:0	0.063 ± 0.003^{a}	0.115 ± 0.021^{b}	0.093 ± 0.020^{ab}	0.009
20:1n-11	0.017 ± 0.002	0.021 ± 0.005	0.017 ± 0.002	0.505
20:1n-9	0.160 ± 0.011	0.144 ± 0.011	0.171 ± 0.026	0.432
20:1n-7	0.006 ± 0.001^{a}	0.015 ± 0.004^{b}	0.009 ± 0.003^{ab}	0.019
20:2n-6	0.062 ± 0.005	0.075 ± 0.008	0.061 ± 0.009	0.223
20:3n-6	0.017 ± 0.002^{a}	0.043 ± 0.006^{b}	0.023 ± 0.002^{a}	0.000001
20:4n-6	0.083 ± 0.007	0.118 ± 0.013	0.103 ± 0.026	0.054
20:3n-3	0.097 ± 0.013	0.088 ± 0.011	0.103 ± 0.023	0.797
20:5n-3	0.028 ± 0.004	0.041 ± 0.005	0.038 ± 0.007	0.084
22:0	0.011 ± 0.002	0.015 ± 0.003	0.025 ± 0.010	0.051
22:1n-9	0.031 ± 0.009	0.059 ± 0.015	0.046 ± 0.014	0.161
22:5n-3	0.056 ± 0.005^{a}	0.103 ± 0.024^{b}	0.103 ± 0.028^{ab}	0.040
24:0	0.037 ± 0.003^{a}	0.070 ± 0.015^{b}	0.060 ± 0.020^{ab}	0.040
22:6n-3 (w/ artefact)	0.387 ± 0.086	1.010 ± 0.370	0.676 ± 0.320	0.123
24:1n-9	0.076 ± 0.005	0.095 ± 0.024	0.129 ± 0.052	0.243
Σ:SFA	68.409 ± 0.613	69.912 ± 0.938	68.177 ± 1.925	0.346
Σ:MUFA	26.493 ± 0.627	24.843 ± 1.027	26.517 ± 1.458	0.267
Σ:PUFA	5.098 ± 0.360	5.245 ± 0.451	5.306 ± 0.985	0.951
Σ:n-6 PUFA	3.103 ± 0.262	2.584 ± 0.193	2.722 ± 0.477	0.303
Σ:n-3 PUFA	1.890 ± 0.161	2.335 ± 0.363	2.423 ± 0.517	0.334
UFA/SFA	0.463 ± 0.013	0.433 ± 0.019	0.472 ± 0.044	0.361
n-3/n-6 PUFA (w/ 22:6n-3)	0.642 ± 0.052	0.923 ± 0.150	0.865 ± 0.086	0.063
n-3/n-6 PUFA (w/o 22:6n-3)	0.501 ± 0.038	0.533 ± 0.058	0.615 ± 0.088	0.379
TACL	16.493 ± 0.020	16.497 ± 0.062	16.502 ± 0.051	0.991
Δ9-DI	0.389 ± 0.012	0.362 ± 0.021	0.393 ± 0.034	0.433
Δ5-DI (n-6 PUFA)	5.657 ± 0.532^{b}	3.110 ± 0.353^{a}	4.271 ± 0.681^{ab}	0.00045
DBI	0.401 ± 0.010	0.413 ± 0.021	0.421 ± 0.033	0.722
Prod/prec (n-6 PUFA)	0.036 ± 0.003^{a}	0.072 ± 0.009^{b}	0.055 ± 0.011^{ab}	0.000057
Prod/prec (n-3 PUFA)	0.357 ± 0.076	1.125 ± 0.430	0.689 ± 0.336	0.083

MMEA = mild/moderate asthma, SEA = severe asthma, *i* = *iso*-methyl-branch, *ai* = *anteiso*-methyl-branch, *c* = *cis*, *t* = *trans*, Σ = sum, SFA = saturated FA, MUFA = monounsaturated FA, PUFA = polyunsaturated FA, UFA = unsaturated FA, TACL = total average chain length, Δ9-DI = delta-9 desaturation index, Δ5-DI = delta-5 desaturation index, DBI = double bond index, prod = product, prec = precursor, different superscript letters indicate significant differences between the means within a row (generalized linear model, $p < 0.05$)

Supplementary table 3. Fatty acid (FA) proportions (mol-%, listed in the order of retention times of chromatographic peaks), sums, and indices in the extracellular vesicles of bronchoalveolar lavage fluid of horses according to the severity of asthma (mean ± SE).

FA/sum/index	Control	MMEA	SEA	*P*
14:0*i*	0.204 ± 0.058[a]	0.189 ± 0.046[a]	0.704 ± 0.266[b]	0.001
14:0	5.113 ± 0.221	5.838 ± 0.563	5.316 ± 0.770	0.389
14:1n-5	0.116 ± 0.030[a]	0.133 ± 0.034[a]	0.348 ± 0.098[b]	0.001
15:0*i*	0.086 ± 0.021	0.073 ± 0.011	0.143 ± 0.042	0.149
15:0*ai*	0.137 ± 0.026[a]	0.136 ± 0.032[a]	0.442 ± 0.164[b]	0.001
15:0	0.347 ± 0.032	0.407 ± 0.062	0.289 ± 0.042	0.290
16:0*i*	0.417 ± 0.044	0.401 ± 0.027	0.520 ± 0.120	0.363
16:0	55.097 ± 1.340[b]	53.231 ± 2.600[b]	43.007 ± 4.578[a]	0.003
16:1n-9	0.386 ± 0.029	0.358 ± 0.034	0.398 ± 0.084	0.781
16:1n-7	5.299 ± 0.404	5.275 ± 0.588	4.914 ± 1.551	0.925
16:1n-5	0.038 ± 0.004[a]	0.041 ± 0.007[a]	0.111 ± 0.040[b]	0.000273
17:0*i*	0.172 ± 0.032[a]	0.235 ± 0.082[a]	0.530 ± 0.163[b]	0.004
17:0*ai*	0.186 ± 0.015[a]	0.176 ± 0.031[a]	0.354 ± 0.111[b]	0.007
17:0	0.329 ± 0.025[a]	0.385 ± 0.052[a]	0.894 ± 0.353[b]	0.001
17:1n-8	0.239 ± 0.018[b]	0.202 ± 0.023[a]	0.315 ± 0.036[c]	0.011
18:0*i*	0.211 ± 0.059	0.225 ± 0.071	0.366 ± 0.196	0.486
18:0	6.907 ± 0.488	8.765 ± 1.539	11.465 ± 3.167	0.072
18:1n-9	15.650 ± 0.591	15.669 ± 1.206	18.228 ± 2.319	0.266
18:1n-7	1.493 ± 0.081	1.562 ± 0.131	1.847 ± 0.271	0.198
18:1n-5	0.173 ± 0.051	0.286 ± 0.110	0.550 ± 0.240	0.055
19:0*i*	0.061 ± 0.011[a]	0.067 ± 0.020[a]	0.145 ± 0.043[b]	0.014
18:2n-6	2.398 ± 0.233	2.026 ± 0.202	1.931 ± 0.322	0.336
19:0	1.864 ± 0.557	1.237 ± 0.397	2.092 ± 0.639	0.567
19:1n-10	0.055 ± 0.011	0.045 ± 0.011	0.065 ± 0.022	0.628
19:1n-8	0.040 ± 0.006	0.042 ± 0.016	0.054 ± 0.026	0.766
18:3n-3	1.121 ± 0.111	0.840 ± 0.068	1.335 ± 0.463	0.152
18:2*c*9*t*11	0.598 ± 0.207	0.760 ± 0.268	1.265 ± 0.639	0.346
20:0	0.089 ± 0.009[a]	0.148 ± 0.037[ab]	0.220 ± 0.060[b]	0.008
20:1n-11	0.017 ± 0.003	0.016 ± 0.002	0.029 ± 0.007	0.052
20:1n-9	0.130 ± 0.012	0.156 ± 0.035	0.150 ± 0.020	0.652
20:1n-7	0.008 ± 0.001[a]	0.011 ± 0.003[a]	0.020 ± 0.004[b]	0.003
20:2n-6	0.069 ± 0.006	0.090 ± 0.024	0.134 ± 0.042	0.071
20:3n-6	0.055 ± 0.011[a]	0.065 ± 0.016[a]	0.122 ± 0.025[b]	0.016
20:4n-6	0.128 ± 0.023	0.122 ± 0.015	0.227 ± 0.068	0.054
20:3n-3	0.084 ± 0.009	0.130 ± 0.064	0.074 ± 0.013	0.540
20:5n-3	0.055 ± 0.011[a]	0.051 ± 0.011[a]	0.133 ± 0.058[b]	0.021
22:0	0.023 ± 0.006	0.021 ± 0.007	0.046 ± 0.017	0.120
22:1n-9	0.058 ± 0.014	0.060 ± 0.011	0.097 ± 0.046	0.380
22:5n-3	0.204 ± 0.056	0.226 ± 0.085	0.425 ± 0.196	0.253
24:0	0.068 ± 0.016[a]	0.062 ± 0.022[a]	0.221 ± 0.071[b]	0.001
22:6n-3 (w/ 22:6n-3)	0.200 ± 0.077	0.161 ± 0.049	0.336 ± 0.178	0.471
24:1n-9	0.079 ± 0.014	0.076 ± 0.018	0.140 ± 0.045	0.110
Σ:SFA	71.309 ± 0.790[b]	71.597 ± 1.129[b]	66.754 ± 1.372[a]	0.008
Σ:MUFA	23.779 ± 0.757	23.932 ± 1.090	27.265 ± 1.523	0.068
Σ:PUFA	4.912 ± 0.375	4.472 ± 0.450	5.982 ± 1.248	0.234
Σ:n-6 PUFA	2.650 ± 0.228	2.303 ± 0.198	2.414 ± 0.416	0.529
Σ:n-3 PUFA	1.663 ± 0.168	1.408 ± 0.149	2.303 ± 0.710	0.107
UFA/SFA	0.405 ± 0.016[a]	0.400 ± 0.023[a]	0.501 ± 0.031[b]	0.0006
n-3/n-6 PUFA (w/ 22:6n-3)	0.664 ± 0.072	0.632 ± 0.072	0.893 ± 0.151	0.156
n-3/n-6 PUFA (w/o 22:6n-3)	0.580 ± 0.053	0.561 ± 0.056	0.779 ± 0.129	0.103
TACL	16.498 ± 0.043	16.493 ± 0.075	16.697 ± 0.155	0.158
Δ9-DI	0.344 ± 0.014[a]	0.342 ± 0.021[a]	0.433 ± 0.028[b]	0.006
Δ5-DI (n-6 PUFA)	2.895 ± 0.352	2.732 ± 0.440	1.875 ± 0.337	0.273
DBI	0.367 ± 0.012[a]	0.356 ± 0.016[a]	0.442 ± 0.033[b]	0.004
Prod/prec (n-6 PUFA)	0.090 ± 0.020[a]	0.099 ± 0.017[a]	0.177 ± 0.026[b]	0.025
Prod/prec (n-3 PUFA)	0.247 ± 0.084	0.273 ± 0.091	0.331 ± 0.071	0.845

MMEA = mild/moderate asthma, SEA = severe asthma, *i* = *iso*-methyl-branch, *ai* = *anteiso*-methyl-branch, *c* = *cis*, *t* = *trans*, Σ = sum, SFA = saturated FA, MUFA = monounsaturated FA, PUFA = polyunsaturated FA, UFA = unsaturated FA, TACL = total average chain length, Δ9-DI = delta-9 desaturation index, Δ5-DI = delta-5 desaturation index, DBI = double bond index, prod = product, prec = precursor, different superscript letters indicate significant differences between the means within a row (generalized linear model, $p < 0.05$)

Supplementary table 4. Grouping of fatty acids derived from the Pearson correlations-based correlogram.

**Group 1**	**Group 2**	**Group 3**	**Group 4**	**Group 5**	**Group 6**
18:2n-6	14:0	16:1n-5	16:0*i*	19:1n-10	19:0
16:1n-9	15:0	18:0*i*	24:1n-9	20:1n-11	19:0*i*
18:3n-3	16:0	22:5n-3	22:6n-3*	19:1n-8	24:0
	16:1n-7	17:0*i*	17:1n-8	20:1n-7	14:1n-5
		18:2*c*9*t*11	15:0*i*	20:2n-6	14:0*i*
		18:1n-5	22:1n-9	20:1n-9	15:0*ai*
		18:0		20:3n-3	20:3n-6
		20:0			22:0
		17:0*ai*			20:4n-6
		17:0			20:5n-3
		18:1n-7			
		18:1n-9			

*plus artefact

Supplementary table 5. Random Forest (RF) prediction accuracy for the different RF strategies and across sample types. Scores represent the averages of 20 leave-one-out RF runs.

**Analysis type/Sample type**	**All samples**	**BALF**	**SUP**	**EVs**
FA data, grouping, enrichment	0.44	0.43	0.47	0.40
FA and DI data, grouping, enrichment	0.48	0.46	0.57	0.43
FA and DI data, grouping, enrichment, DI excluded from RF model	0.49	0.42	0.59	0.41
FA data	0.48	0.35	0.55	0.39

FA = fatty acid, DI = derived structural category sums, ratios, and indices of FAs, BALF = bronchoalveolar lavage fluid, SUP = BALF supernatant, EV = extracellular vesicles. The derived sums, ratios, and indices were as follows: Δ5-desaturation index, Δ9-desaturation index, Σ:SFA (saturated fatty acid), Σ:MUFA (monounsaturated fatty acid), Σ:PUFA (polyunsaturated fatty acid), Σ:n-3 PUFA, Σ:n-6 PUFA, double bond index, n-3/n-6 PUFA ratio (both with and without 22:6n-3), product/precursor ratios of n-3 PUFAs, product/precursor ratios of n-6 PUFAs, UFA (unsaturated fatty acid)/SFA ratio, total average chain length.

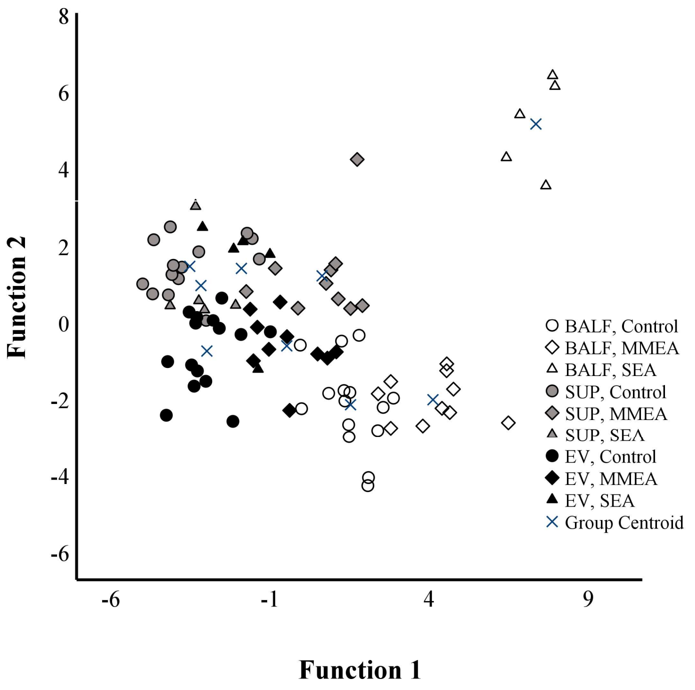

Supplementary figure 1. Discriminant analysis with 9 groups (bronchoalveolar lavage fluid = BALF, supernatant = SUP, and extracellular vesicles = EVs each assigned into either control, mild/moderate equine asthma = MMEA, or severe equine asthma = SEA), depicting the classification of fatty acid data in equine BALF, SUP, and EVs of control horses and horses with MMEA or SEA based on discriminant functions 1 (on x-axis) and 2 (y-axis). White symbols = BALF, grey symbols = SUP, black symbols = EVs. See Results for details.

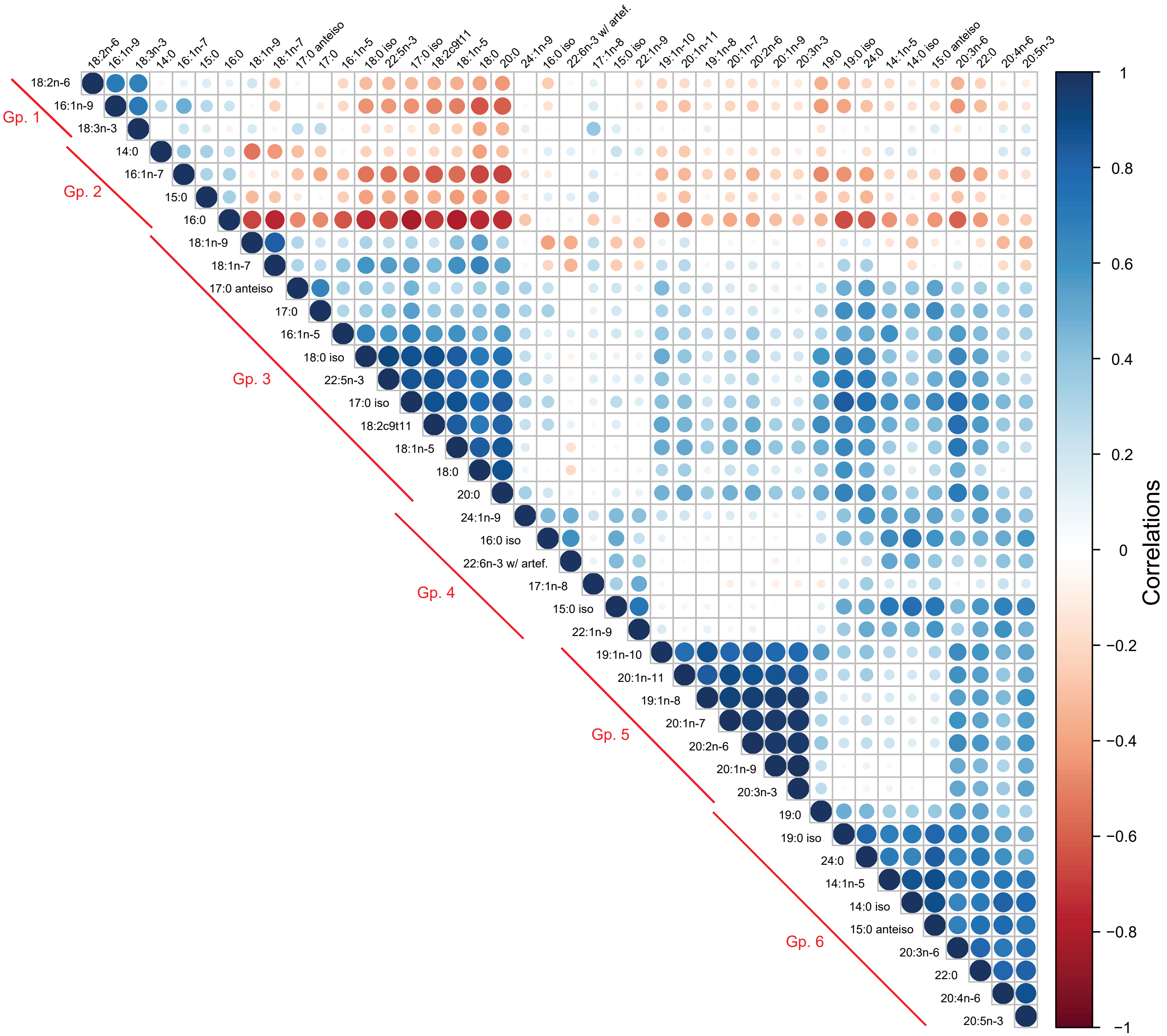

Supplementary figure 2. Correlogram showing the Pearson correlation coefficients between pairs of fatty acids (FAs) across all samples, color-coded as indicated. Dark blue (respectively red) indicates strong positive (respectively negative) correlations in FA levels, irrespective of the health status. Groups (Gp.) of FAs identified with hierarchical clustering are indicated with red bars on the left. Correlogram was plotted using the Corrplot function in R.

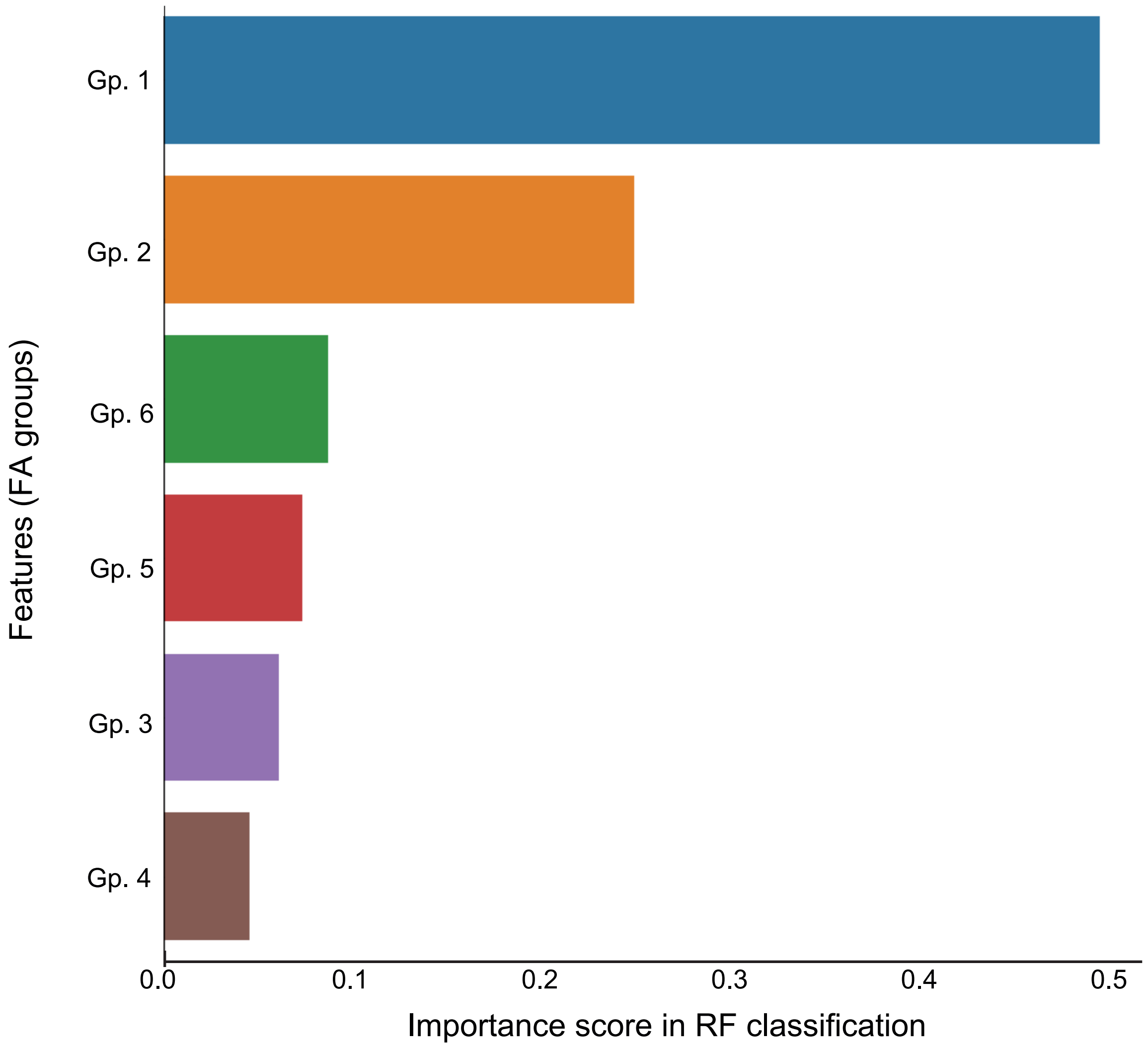

Supplementary figure 3. Random forest (RF)-based classification of the samples' inflammatory status. Bar chart showing the feature importance score of each fatty acid (FA) group (Gp.) in the RF-based classification of samples' inflammatory state (as diagnosed). Shown scores are averages of the FA group importance scores over 100 RFs of 100 trees each, run on a 1000-fold enriched dataset (see Materials and Methods for details).

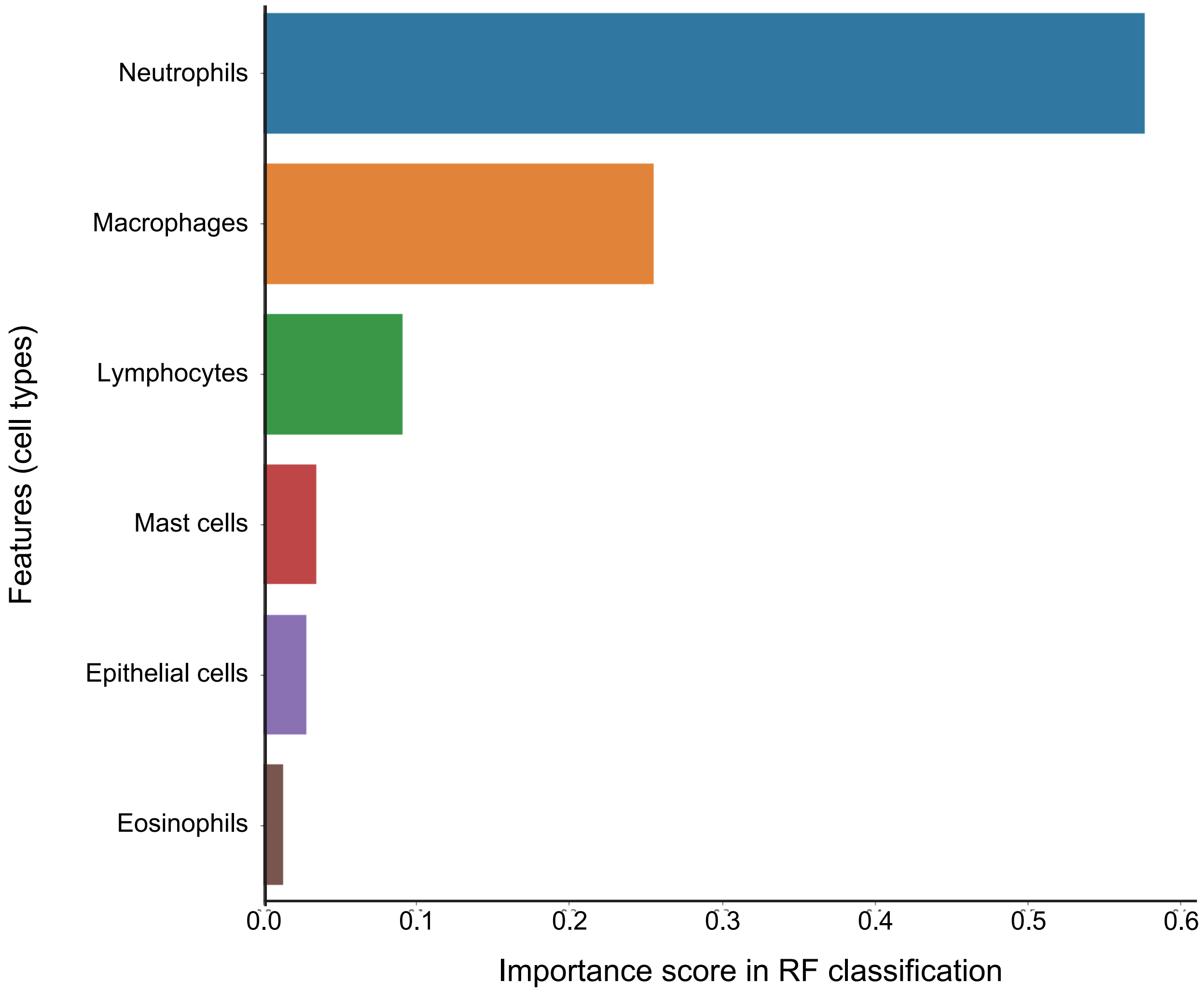

Supplementary figure 4. Immune cells in Random forest (RF)-based classification. Bar chart showing the feature importance score of each cell type in the RF-based classification of samples' inflammatory state based on the bronchoalveolar lavage fluid cell-%. Shown scores were obtained using 1 RF of 100 trees, without data enrichment.

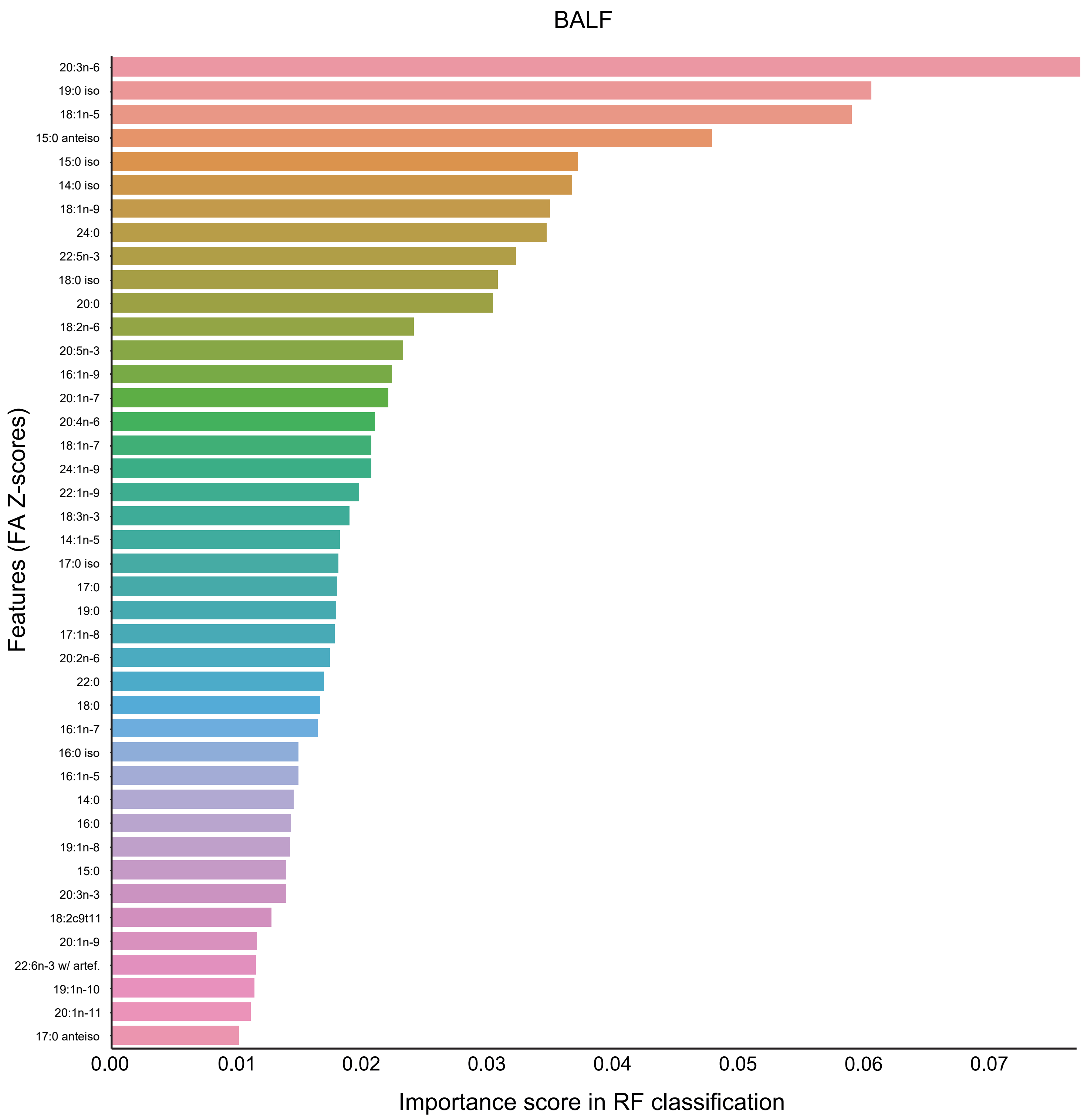

Supplementary figure 5. Random forest (RF)-based classification of diagnoses in bronchoalveolar lavage fluid (BALF) samples. Bar chart showing the feature importance score of each fatty acid (measured FA abundance converted to a Z-score) in the RF-based classification of samples' inflammatory state. Shown scores were averaged over 100 RFs of 100 trees, without data enrichment.

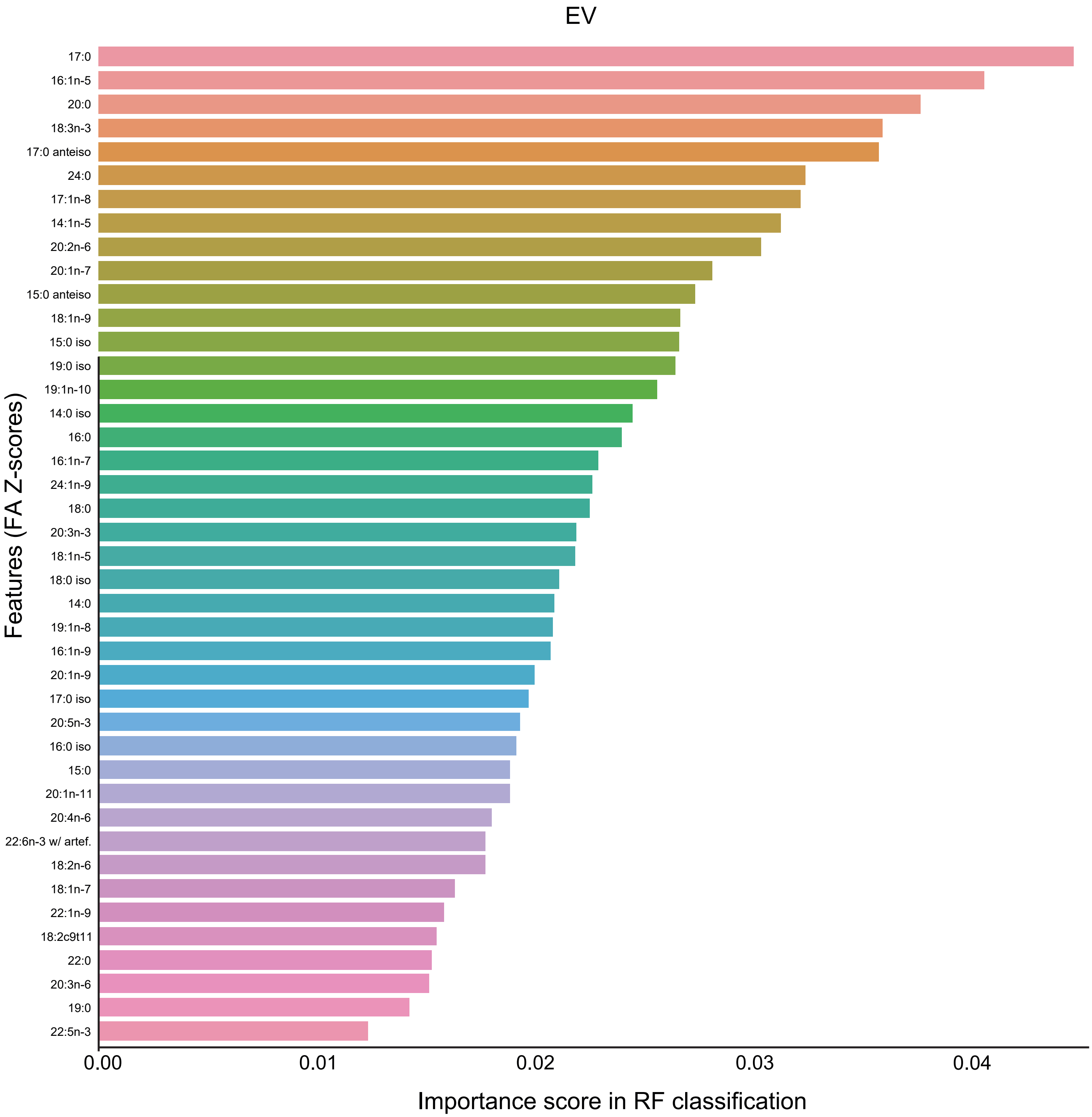

Supplementary figure 6. Random forest (RF)-based classification of diagnoses in extracellular vesicle (EV) samples. Bar chart showing the feature importance score of each fatty acid (measured FA abundance converted to a Z-score) in the RF-based classification of samples' inflammatory state. Shown scores were averaged over 100 RFs of 100 trees, without data enrichment.
